# Nitration Mechanism of Aromatics: Lessons from Born–Oppenheimer Molecular Dynamics

**DOI:** 10.1021/acsphyschemau.5c00086

**Published:** 2025-11-17

**Authors:** Fabio J. F. S. Henrique, Pierre M. Esteves

**Affiliations:** Instituto de Química, 28125 Universidade Federal do Rio de Janeiro, Av. Athos da, Silveira Ramos, 149, CT, A-622, Cid. Univ., Rio de Janeiro, 21941-909, RJ, Brazil

**Keywords:** nitration, electrophilic, aromatic substitution, Born−Oppenheimer molecular dynamics, superelectrophile

## Abstract

The nitration of aromatic compounds is a fundamental transformation in organic chemistry, traditionally understood through the Ingold–Hughes polar mechanism and, more recently, via single-electron transfer (SET) pathways. In this work, Born–Oppenheimer molecular dynamics (BOMD) simulations were employed to explore the mechanistic features of toluene nitration in a protic polar medium, specifically a concentrated sulfonitric mixture (HNO_3_/H_2_SO_4_). Simulations at 423 K revealed the spontaneous formation of the nitronium ion (NO_2_^+^) via double protonation of HNO_3_ by H_2_SO_4_. Several BOMD trajectories were analyzed for the reaction between toluene and NO_2_^+^ at 300 K, leading to four different reaction outcomes: (i) no reaction, highlighting nucleophilic rather than protic solvation of NO_2_^+^; (ii) nitration at the positions *ortho* and *para* via a V-shaped [NO_2_·ArH]^+^ SET complex evolving into a σ-complex and ultimately the *o*- or *p*-nitrotoluene after deprotonation; (iii) oxygen transfer resulting in *o*-cresol and NO, initiated from a Λ-shaped [NO_2_·ArH]^+^ SET complex; and (iv) the formation of a cyclohexadienone–NO complex via 1,2-hydride shift, also proceeding through a Λ-shaped [NO_2_·ArH]^+^ intermediate. Electronic structure analyses (HOMO/LUMO, spin density, Bader charges) confirmed SET as the key step in all reacting pathways. No evidence of superelectrophilic solvation was observed under BOMD conditions. These results reinforce the role of SET in electrophilic aromatic nitration under strongly acidic conditions and reveal new oxygen transfer pathways dependent on the spatial orientation of the NO_2_^+^ relative to the aromatic ring.

## Graphical abstract

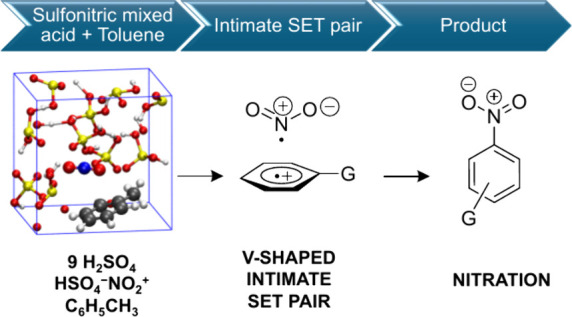

## Introduction

Organic chemistry evolved from the classificatory organization by functional groups (a product of the 19th-century chemistry) to the organization according to mechanistic classes, which was proposed mainly, but not exclusively, by Ingold.^1^ One of the main classes in that scope is the electrophilic aromatic substitution. Among these reactions, electrophilic aromatic nitration reactions are important,^2^ having been one of the first reactions of this type reported, when Faraday reported the aroma of almonds upon mixing benzene, which he had previously isolated, with nitric acid.^3^ Actually, in 2025, we celebrated the 200th anniversary of the discovery of benzene by Michal Faraday, who first isolated and identified benzene in 1825 from the oily residue derived from the production of illuminating gas.^3,4^

The mechanism of electrophilic aromatic nitration has been debated for decades, revealing interesting secrets from a mechanistic point of view. Ingold and Hughes proposed that the nitronium ion (NO_2_^+^) acts as the reactive electrophile, forming an arenium ion intermediate (ArXNO_2_^+^, also known as a σ-complex or Wheland intermediate). This intermediate eventually undergoes deprotonation, affording the neutral nitrated product. This is known as the Ingold-Hughes mechanism. Kerner^5^ and Weiss^6^ suggested an alternative single-electron transfer (SET) mechanism, where the aromatic radical cation (ArH^+•^) and the ^•^NO_2_ radical recombine to form the same intermediate, ArHNO_2_^+^. Additional support for this latter mechanism comes from several experimental and theoretical contributions,^7,8^ although there is some debate about it.^9–11^ A mechanistic continuum involving the polar mechanism and single-electron transfer (SET) has been proposed, as shown in Figure 1.^12–14^

**Figure 1:**
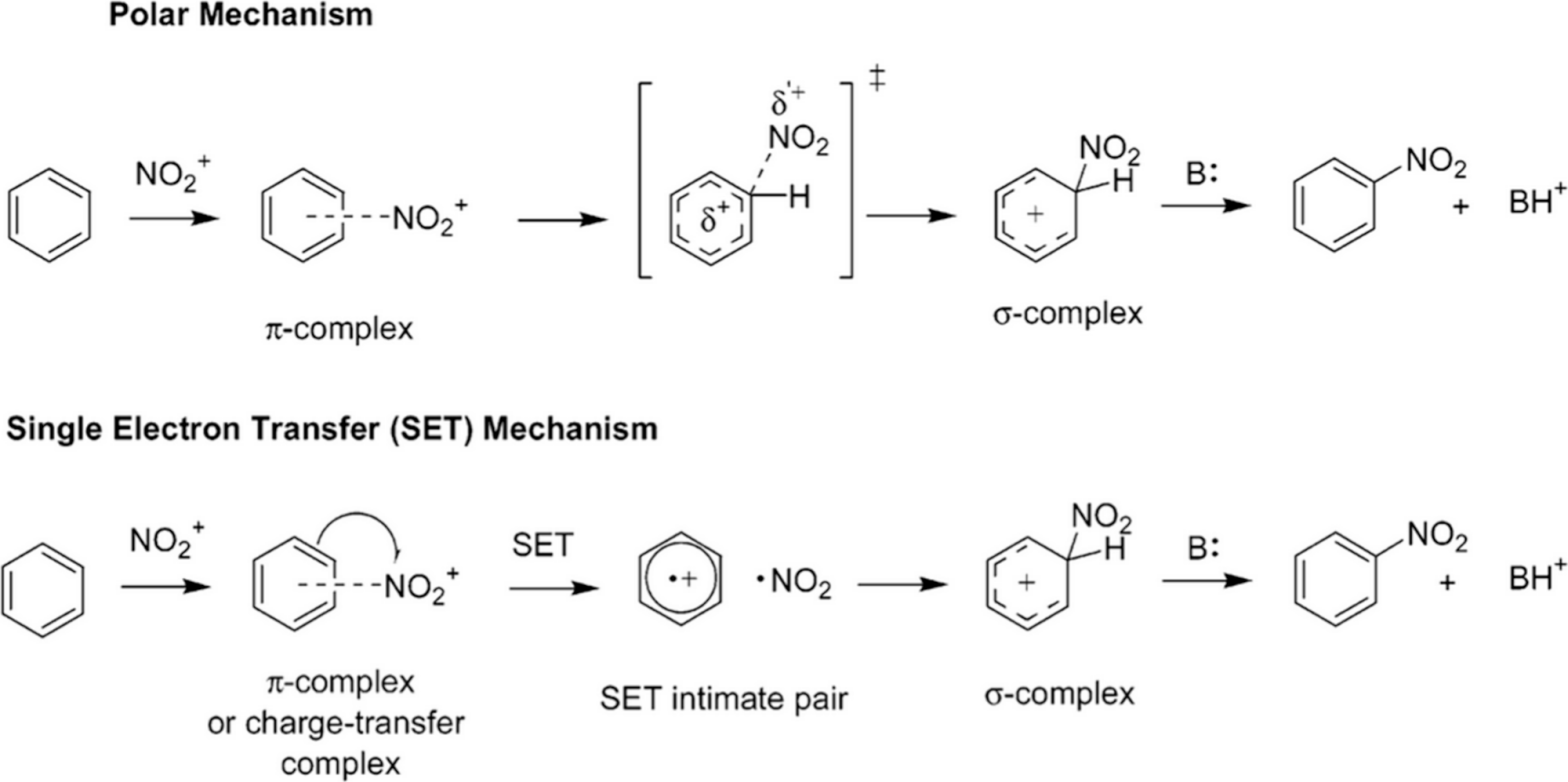
Mechanistic continuum previously proposed for the nitration of aromatic compounds. Reproduced from ref (^13^). Copyright 2006 American Chemical Society.

The solvent can play an important role in determining the outcome of the reaction.^13,15^ Olah suggested^16^ that the nitration of some aromatics could occur not with the NO_2_^+^ species as the active electrophilic species, but, instead, by the proton nitronium dication [NO_2_H]^2+^, i.e. the dication formed by the protonation of the NO_2_^+^, as part of a concept known as supereletrophilic solvation.^17–19^ There are other discussions about the solvent role in aromatic nitration. The nitration of toluene by NO_2_^+^. BF_4_^–^ in CH_2_Cl_2_ with explicit solvation was investigated by both Singleton^15^ and Peluso^20^ using molecular dynamics calculations. The authors found that toluene nitration by NO_2_BF_4_ in dichloromethane is accurately predicted only with explicit solvent and counterion in trajectory computations, while transition state theory fails to account for selectivity. Peluso et al. showed that a SET step is systematically involved in every reactive trajectory.^20^ Gas-phase mass spectrometric studies of the reactions of the naked (NO_2_^+^) and monosolvated (CH_3_NO_2_·NO_2_^+^) nitronium ion with various monosubstituted aromatic compounds, supported by theoretical calculations, indicated that a general model for regioselectivity that relies on the single-electron transfer (SET) mechanism and solvation may have an important role.^9,13^

This interesting discussion about aromatic nitration still lacks a study considering a strongly polar protic reaction medium, such as the mixture of concentrated HNO_3_ and concentrated H_2_SO_4_, also known as the sulfonitric mixture, which is commonly used in such reactions. Herein, we describe the results of Born–Oppenheimer molecular dynamics (BOMD) simulations of the nitration of model aromatic compounds in the sulfonitric mixture, considering explicit dynamics and solvation in these strongly protic reaction media, commonly used for aromatic nitration. This study aimed to add to the understanding of the nitration of aromatics mechanism and the role of solvation in this process.

### Computational Details

To investigate the mechanism of aromatic nitration, two complementary phenomena were explored: (1) the generation of the electrophilic species in a concentrated mixture of nitric and sulfuric acids (commonly referred to as the sulfonitric mixture), which acts as the reactive medium for electrophilic aromatic substitution; and (2) the subsequent interaction between the solvated electrophile and a model aromatic compound (toluene). The initial system, designed to model the formation of the electrophile, consisted of ten molecules of sulfuric acid (H_2_SO_4_) and one molecule of nitric acid (HNO_3_), placed in a cubic simulation box with an edge length of 10 Å, which corresponds to the approximate density of concentrated sulfuric acid under ambient conditions (Figure 2a). This configuration enabled the spontaneous formation of the nitronium ion (NO_2_^+^), a well-known electrophile in nitration processes.^21–23^ To simulate the reactivity toward an aromatic substrate, the system was subsequently modified to include nine molecules of sulfuric acid, one nitronium ion stabilized by a hydrogen sulfate counterion (HSO_4_^–^), and one molecule of toluene, all within the same simulation cell dimensions (Figure 2b).

**Figure 2:**
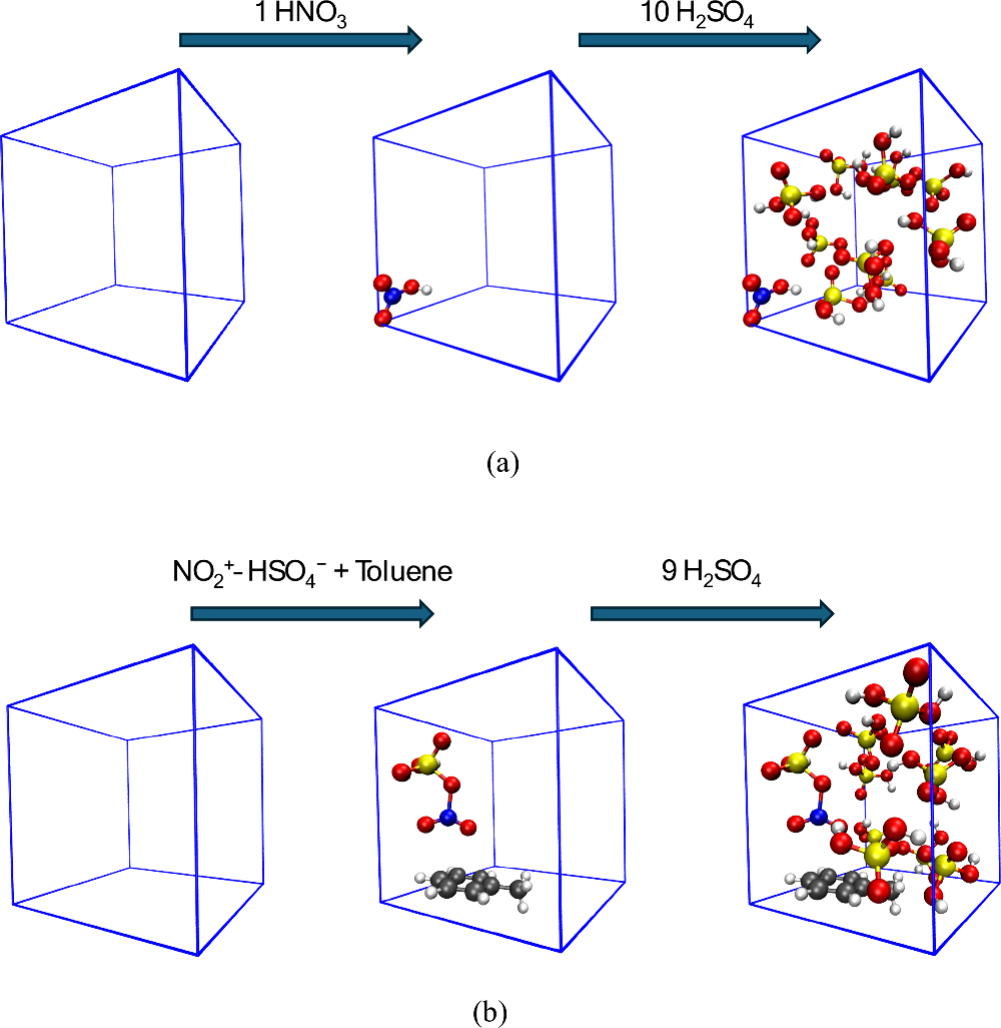
Schematic representation of (a) the sulfonitric mixture and (b) a typical initial configuration consisting of nine molecules of sulfuric acid, one nitronium ion stabilized by a hydrogen sulfate anion, and one toluene molecule confined within a cubic simulation box with an edge length of 10 Å. The molecules are homogeneously distributed throughout the volume. Several different initial configurations were considered for the BOMD calculations. Atom color scheme: gray = carbon, red = oxygen, blue = nitrogen, white = hydrogen, yellow = sulfur.

Born–Oppenheimer molecular dynamics (BOMD) simulations were performed using the CP2K version 2025.1 package, with the Quickstep module providing an efficient implementation of density functional theory (DFT) based on the Gaussian and Plane-Wave (GPW) approach.^24,25^ The unrestricted Kohn–Sham (UKS) formalism was employed to account for spin polarization that allows for radical formation during the simulations, if this is the case. The exchange–correlation energy was treated using the Perdew–Burke–Ernzerhof (PBE)^26^ functional within the generalized gradient approximation (GGA), along with Grimme’s D3­(BJ) empirical dispersion correction^27^ to incorporate van der Waals interactions.

Core electrons were described using Goedecker–Teter–Hutter (GTH) pseudopotentials,^28^ while valence electrons were expanded using Dunning’s cc-pVDZ basis set.^29^ A plane-wave cutoff of 600 Ry and a relative cutoff of 60 Ry were applied, with a multigrid scheme using four levels. The self-consistent field (SCF) procedure employed the orbital transformation (OT) method^30^ and a convergence criterion of 1 × 10^–6^ Hartree.

All BOMD simulations were conducted in the canonical (NVT) ensemble at fixed temperatures using the canonical sampling through velocity rescaling (CSVR) thermostat^31^ with a time constant of 0.1 fs. A time step of 0.5 fs was adopted, and simulations were carried out in a 10 × 10 × 10 Å^3^ cubic box with periodic boundary conditions in all directions. Two sets of simulations were performed: in the first set, the thermal behavior of the sulfonitric mixture (without toluene) was analyzed at 300 and 373 K, using 25000 steps (12.5 ps), and at 423 K using 50000 steps (25 ps).

To evaluate the formation of the nitronium ion in a sulfonitric mixture with a 10:1 molar ratio of H_2_SO_4_ to HNO_3_, a system containing 10 molecules of H_2_SO_4_ and 1 molecule of HNO_3_ was placed in a cubic box of 10 Å.

In the second set of calculations, toluene was added to the sulfonitric mixture, and BOMD simulations occurred at 300 K, with the number of steps and simulation time adjusted to ensure complete evolution and stabilization of the reaction products. The various initial configurations of the system were generated using the Packmol program,^32^ followed by the construction of the unit cell for periodic calculations with a size sufficient to approximately match the density of the sulfonitric mixture, and with different relative orientations of toluene and the nitronium ion. These initial configurations were then thermalized using BOMD. After thermalization, production runs were obtained, typically with 50000 steps of 0.5 fs (totaling 25 ps of BOMD simulation per trajectory).

To gain further insight into the electronic structure and reactivity of the system, representative frames from the MD trajectories were subjected to single-point DFT calculations using a more costly hybrid GGA functional containing Hartree–Fock exchange, namely the PBE0 functional with 49.5% of Hartree–Fock functional and the DZVP-MOLOPT-SR-GTH basis set and the GTH-PBE pseudopotential, available at the CP2K program, to check the HOMO/LUMO orbitals, spin density, and Bader atomic charges. The SCF cycle was performed with a tighter convergence threshold of 1 × 10^–7^ Hartree in the outer loop. Electronic properties, including HOMO–LUMO orbitals, total electron density, and spin density, were calculated for subsequent visualization and analysis.

## Results and Discussion

Figure 3a shows the angle of the NO_2_ group and the distance between the NO_2_ group and the hydroxyl group of HNO_3_. During the first 8 ps of the simulation, no changes were observed, with HNO_3_ remaining stable within the solvation shell composed of H_2_SO_4_, as shown, for example, in Figure 3b for the 2 ps frame (step 4000). At 8.5 ps, the first protonation of HNO_3_ was observed, where a change in the NO_2_ moiety angle was noticed, averaging 150°, as shown in Figure 3c. This corresponds to the formation of the OH-protonated nitric acid, [H_2_O-NO_2_]^+^, which lasts stable for ∼ 7 ps, when a second protonation occurs at 15.5 ps, forming the diprotonated HNO_3_, [H_3_O-NO_2_]^2+^, a gitonic dication, which immediately decomposes into a hydronium ion and NO_2_^+^ (Figure 3d), being more related to a transition state instead of a long living reaction intermediate. This can likely be due to the repulsion between the two positive charges, leading to a Coulombic explosion. Despite this separation (∼5 Å), the nitronium ion angle decreased to an average of 135°, due to effective ionic interactions with the conjugate base HSO_4_^–^, which helped stabilize it, as shown in Figure 4d. This interaction between NO_2_^+^ and HSO_4_^–^ was interrupted at 22 ps, as seen in Figure 3e, when the nitronium ion reached an angle of 170°, close to its expected angle (180°), due to the protonation of the conjugate base by another acid in the medium. However, the nitronium ion quickly found another conjugate base, reestablishing ionic interactions with the environment and returning to the average angle of 135°. Scheme 1 summarizes graphically this step of the study.

**Figure 3:**
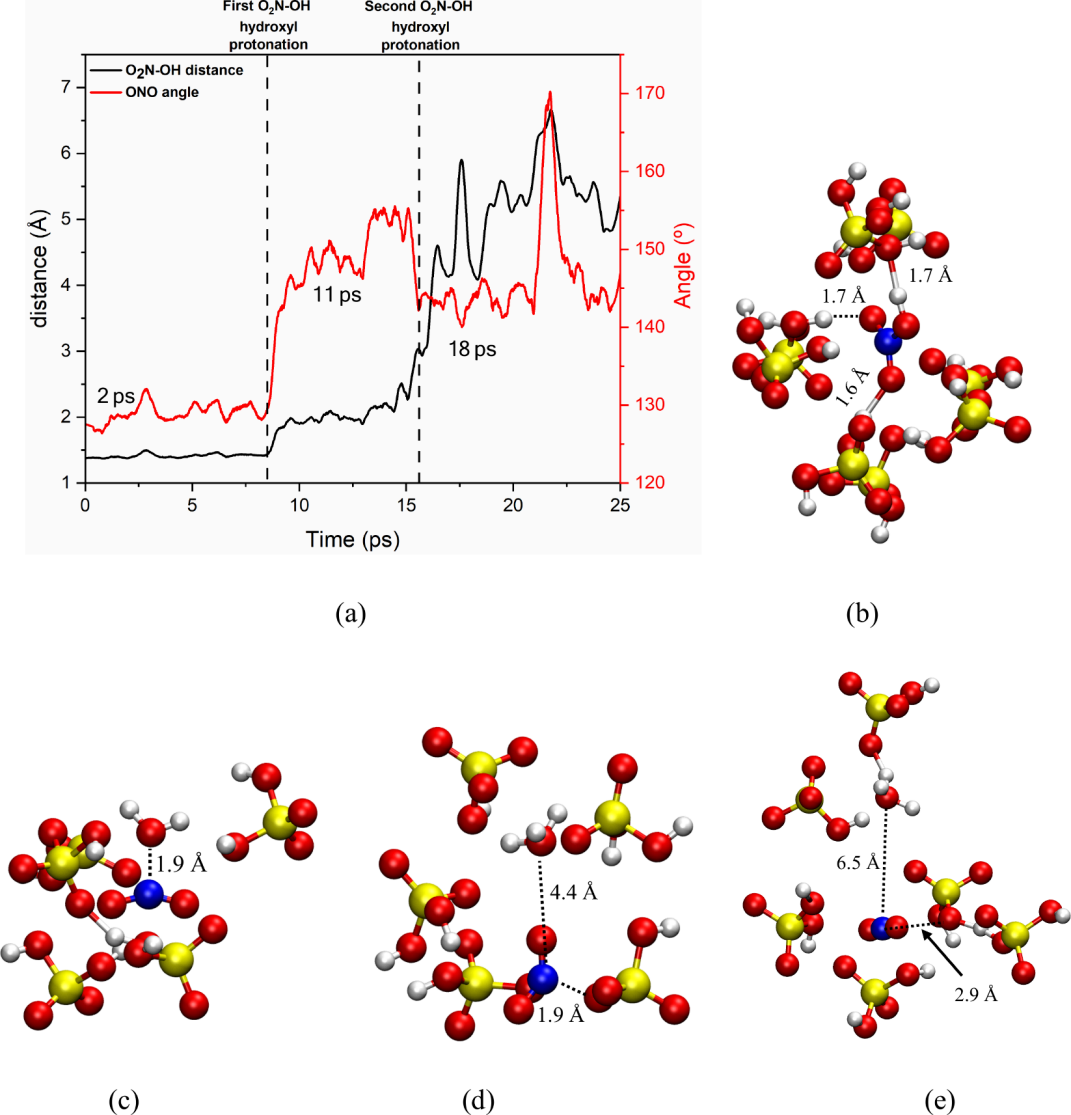
(a) HO–NO_2_ distance and ONO angle as a function of time and solvation sphere with a 4 Å radius around nitric acid at frames (b) 2 ps, (c) 11 ps, (d) 18 ps, and (e) 22 ps from the simulation of the sulfonitric mixture at 423 K. Atom colors: red = oxygen, yellow = sulfur, blue = nitrogen, and white = hydrogen. Interatomic distances are given in angstroms.

**Scheme 1:**
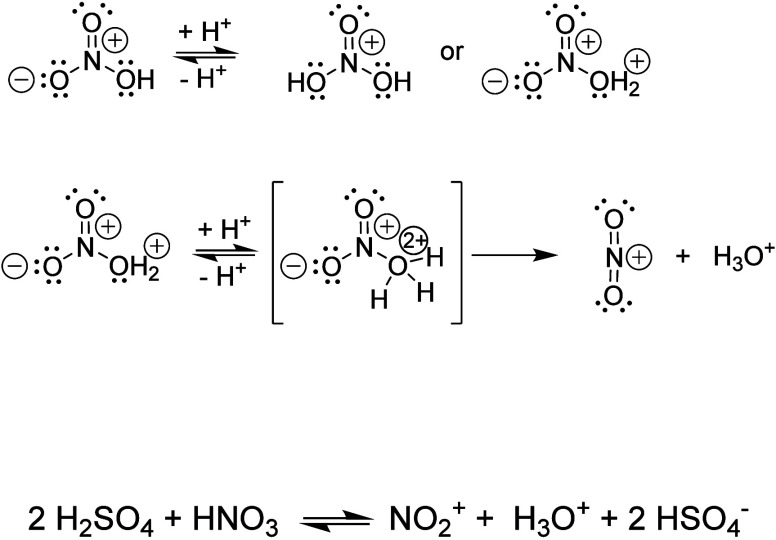
Proposed Mechanism for the Formation of the Nitronium Ion in a Sulfonitric Mixture

**Figure 4:**
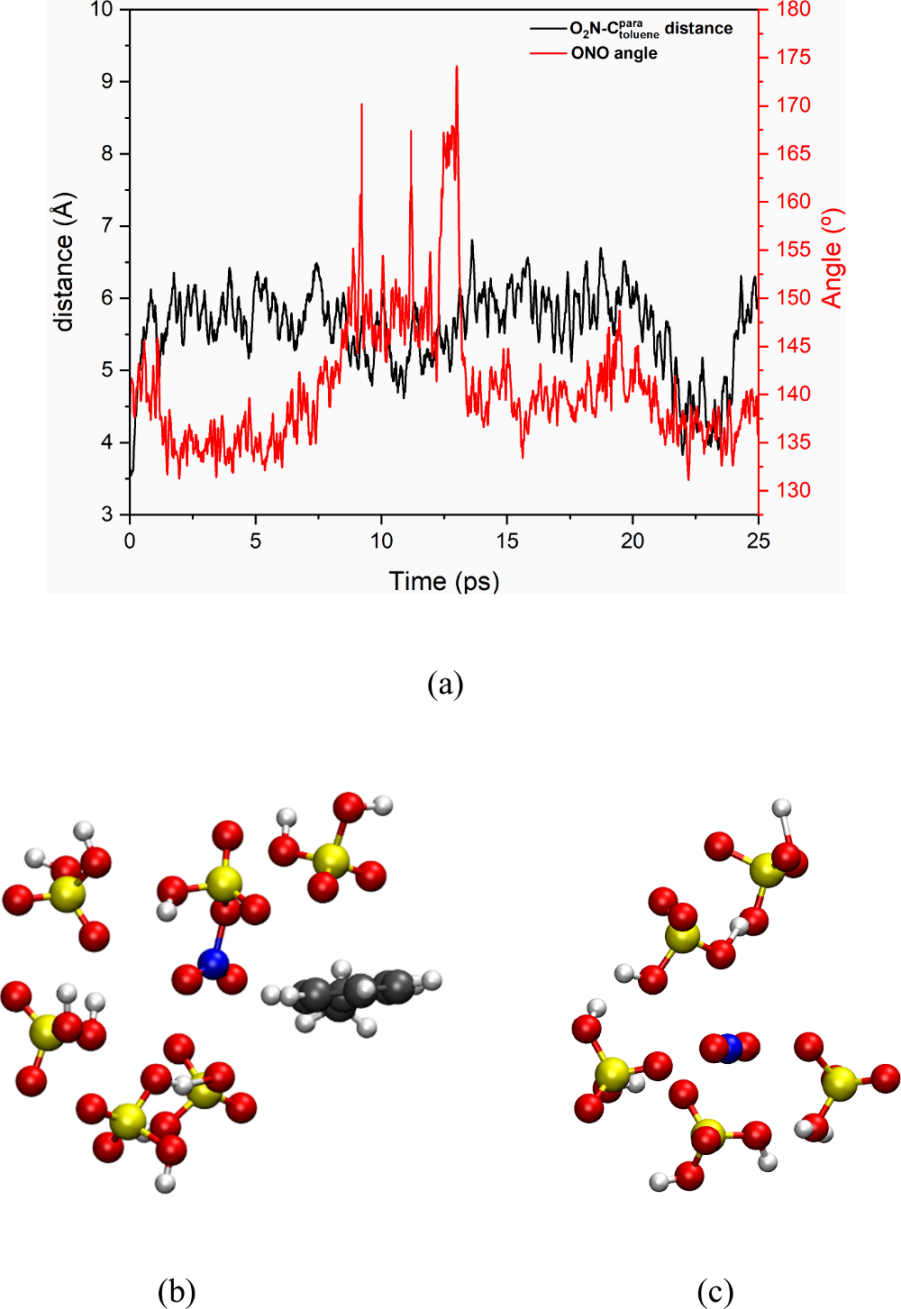
(a) N_(NO2)_/^para^C_toluene_ distance and ONO angle as a function of time and solvation sphere with a 4 Å radius around nitric acid at selected frames illustrating (b) the typical bonded O_2_N-OSO_3_H intermediate and (c) the NO_2_^+^ ion formed upon protonation of O_2_N-OSO_3_H intermediate, in of the sulfonitric mixture at 300 K. Atom colors: red = oxygen, yellow = sulfur, blue = nitrogen, gray = carbon, and white = hydrogen.

With the formation of the nitronium ion confirmed by the BOMD calculations, another system comprising the nitronium ion and a conjugate base (HSO_4_^–^), along with 9 H_2_SO_4_ molecules, was placed in a 10 Å cubic box together with a toluene molecule, considering different initial configurations, to study the nitration of toluene in a sulfonitric mixture. In most of the initial configurations, the toluene was positioned within the solvation sphere of the nitronium ion, as previously shown by Peluso et al.^20^ that both toluene and the nitronium ion must be within the same solvation sphere for the reaction to occur. All calculations involving the sulfonitric mixture with toluene present were carried out at 300 K.

A total of 14 different trajectories starting from different initial conditions (positions, configurations, and velocities) were obtained from the BOMD simulations involving toluene, each initiated from distinct initial configurations and with durations sufficient to allow the formation of stable products. The outcomes of these simulations can be categorized into four distinct reaction pathways. The trajectory movies and raw data corresponding to each of these four outcomes, as well as the trajectory of the sulfonitric mixture at 423 K that resulted in the formation of the nitronium ion, are provided in the repository indicated in the Data Availability statement (see infra) and in the Supporting Information. From the 14 initial different trajectories, 1 resulted in no reaction, 4 led to the formation of the nitration products (2 at the *para* position and 2 at the ortho position), 4 for the intermediates that did not evolve to the final products, and 3 resulted in oxygen transfer to the aromatic ring. Throughout the text, specific frames of selected trajectories will be shown to illustrate typical features observed from the different trajectories. The raw trajectories and related data are available for all systems (Data Availability statement), which will allow the interested reader to analyze/inspect for him-/herself the details of every calculation. Supporting Information contains some selected videos.

### First Group: Nonreacting Trajectories

In the first set of simulations of toluene nitration in a sulfonitric mixture, no nitration reaction was observed, despite toluene being about 3 Å apart from the nitronium ion. These nonreacting trajectories indicate that some preferential relative orientation of the NO_2_^+^ moiety in relation to toluene is necessary, so that some reaction takes place (see infra). This group of simulations offers the possibility of analysis of the solvation dynamics of the reactants. Figure 4a shows the variation in the angle of the nitronium ion and the distance between the nitrogen atom of the nitronium ion and the ortho carbons of toluene as a function of time, over a 25 ps simulation average. The solvation of the NO_2_^+^ ion in these highly acidic and protic solvents is particularly interesting to be analyzed, aiming to find evidence for the so-called protossolvation,^17^ since this kind of solvation has been previously proposed to have a key role in this kind of system.

It was observed that the distance between the nitronium ion and toluene remained stable throughout the entire simulations. Figure 4b presents a typical solvation sphere of the nitronium ion with a radius of 4 Å, in the presence of toluene. In these cases, the interaction between the nitronium ion and toluene occurred through the lateral regions of both molecules, which does not favor a reaction between them. Figure 4c again shows the 4 Å solvation sphere of the nitronium ion, revealing that the toluene molecule moved out of this interaction region and remained outside it until the end of the simulations. Furthermore, analysis of the angular variation of the nitronium ion during the simulation of a typical trajectory (Figure 4a). In general, the NO_2_^+^. HSO_4_^–^ complex remained stable throughout the simulation, with the formation of a free NO_2_^+^ intermediate (ONO angle close to 180°), occurring only in rare events (spikes in the red curve in Figure 4a).

In the simulation where no nitration reaction took place, it was possible to perform an analysis of the solvation of the nitronium ion in H_2_SO_4_ in the presence of toluene. A radial distribution function (RDF) analysis was carried out between the oxygen atoms of the nitronium ion, and all hydrogen atoms present in the system, considering the 50000 frames (typical trajectory length) obtained over the 25 ps long simulations. The RDF plot shown in Figure 5 indicates that the first peak in the distance between the oxygen atoms of the nitronium ion and the hydrogen atoms in the medium occurs at 3.2 Å, much longer than hydrogen bonding (∼1.7 Å) or explicit protonation of NO_2_^+^ (should afford RDF at ∼ 1 Å) as required by superelectrophile formation or superelectrophilic solvation. This result demonstrates that, during the simulation, no hydrogen bonding formation involving the nitronium ion is observed, nor was there any protonation of the oxygen atoms to form the protonitronium dication. This superelectrophilic solvation has been previously proposed by Olah for nitration^17,33^ Thus, the results from the BOMD did not support the protonation or protosolvation of the NO_2_^+^. Therefore, the interactions of the nitronium ion with the H_2_SO_4_ medium occur exclusively through a nucleophilic solvation, with ionic interactions between the nitronium ion and the negatively charged oxygen atoms in the conjugate base HSO_4_^–^ or H_2_SO_4_.

**Figure 5:**
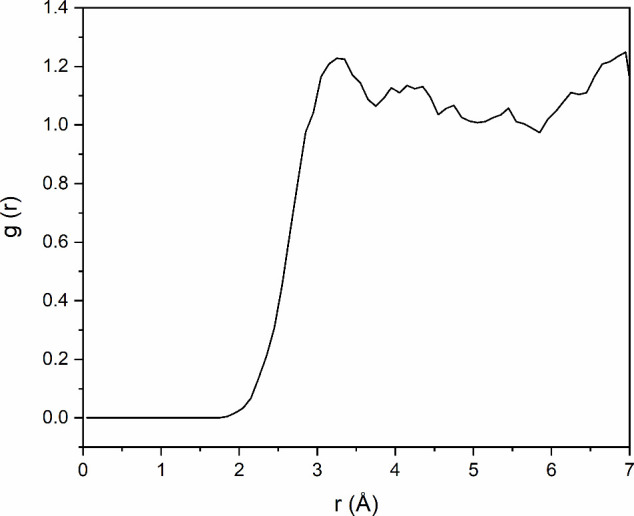
Radial distribution function (RDF) analysis between the oxygen atoms of the nitronium ion and all hydrogen atoms present in the system.

### Reacting Trajectories: Toluene Nitration

The second set of trajectories (4 different trajectories, from different initial configurations) simulations involving the sulfonitric mixture and toluene evolved to nitration products, resulting in the formation of both *para*- and *ortho*-nitrotoluene. No nitration at meta positions was observed in any of the trajectories investigated.

#### Trajectories Leading to the Attack at the Para Position

Figure 6 illustrates the ONO bond angle and the distance between the nitronium group and the carbon at the *para* position of toluene throughout the simulation.

**Figure 6:**
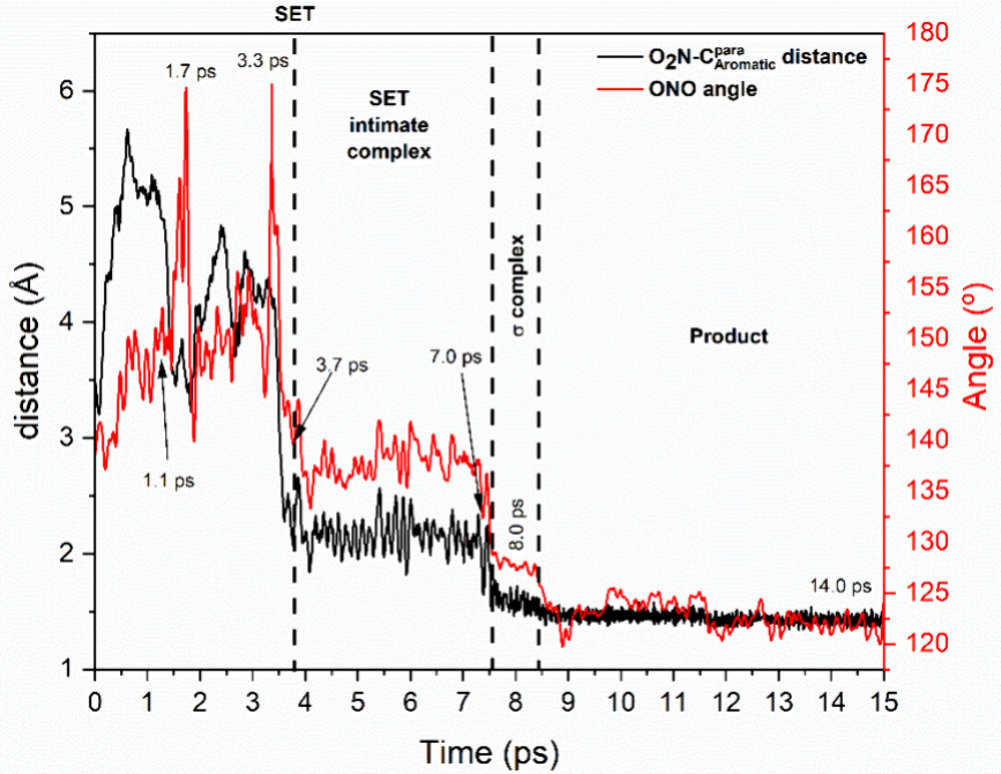
Time evolution of the distance between the NO_2_ group and the carbon atom at the *para* position of toluene of a typical selected trajectory, and the ONO bond angle of the nitronium ion during the nitration process, at 300 K.

At the beginning of the simulations, similarly to what was observed in the formation of the nitronium ion in the sulfonitric mixture, the NO_2_^+^ group remained stabilized by the conjugate base HSO_4_^–^. In Figure 7a, it is evident that the nitrogen atom points toward the oxygen atom of the HSO_4_^–^. In this particular trajectory, two peaks were observed in the ONO angle around 175°, corresponding to rare protonation events of the conjugate base that temporarily stabilize a linear NO_2_^+^ ion, as shown in Figures 7b and 7c (at 1.7 and 3.3 ps, respectively). This is a trend observed in the other trajectories as well.

**Figure 7:**
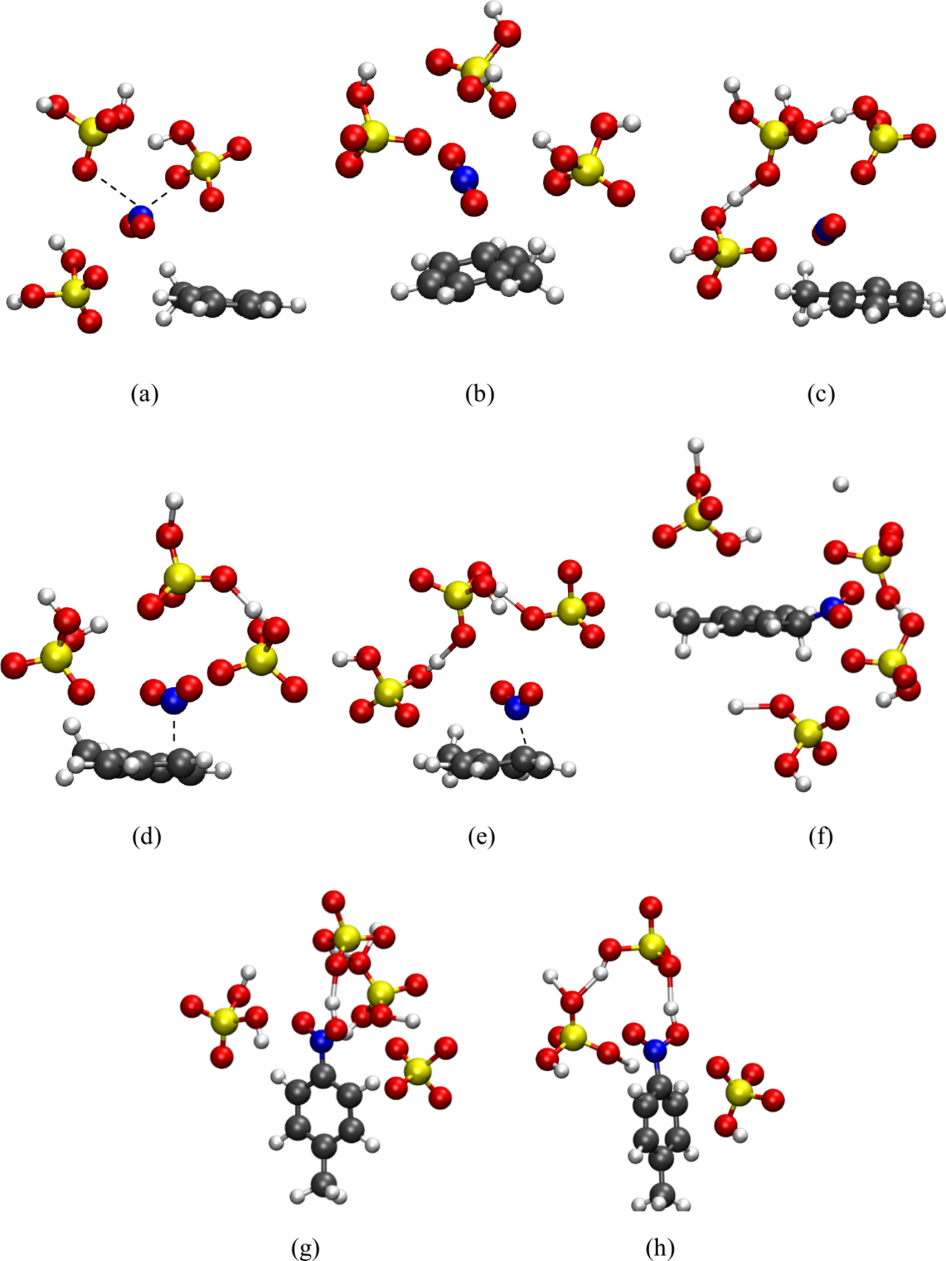
Solvation sphere with a 4 Å radius around the nitronium ion at selected time frames from the toluene nitration simulation at 300 K: (a) 1.1 ps, (b) 1.7 ps, (c) 3.3 ps, (d) 3.7 ps, (e) 7.0 ps, (f) 8.0 ps, (g) 11.9 ps, and (h) 14.0 ps. Atom color scheme: red = oxygen, yellow = sulfur, blue = nitrogen, gray = carbon, white = hydrogen.

In these trajectories, it is observed that a sudden change in the NO_2_^+^ angle is observed (Figure 7d), due to a single electron transfer (SET) event (see infra). In this process, the nitronium ion accepts an electron from toluene, resulting in this bending. Following this SET step, the nitrogen atom rapidly approached the *para*-position of toluene, forming a SET intimate pair (Figure 7e), which survived for approximately 4–60 ps range on average, with the NO_2_ moiety assuming a V-shaped form in [NO_2_.ArH]^+^ complex. This structure is similar to the ones reported in the gas phase.^12,13^ This intermediate is also formed at other positions of the ring and is long-lived enough to migrate between the positions during the dynamics in some cases. Figure 8 shows the optimized structures of these intermediates for each position.

**Figure 8:**
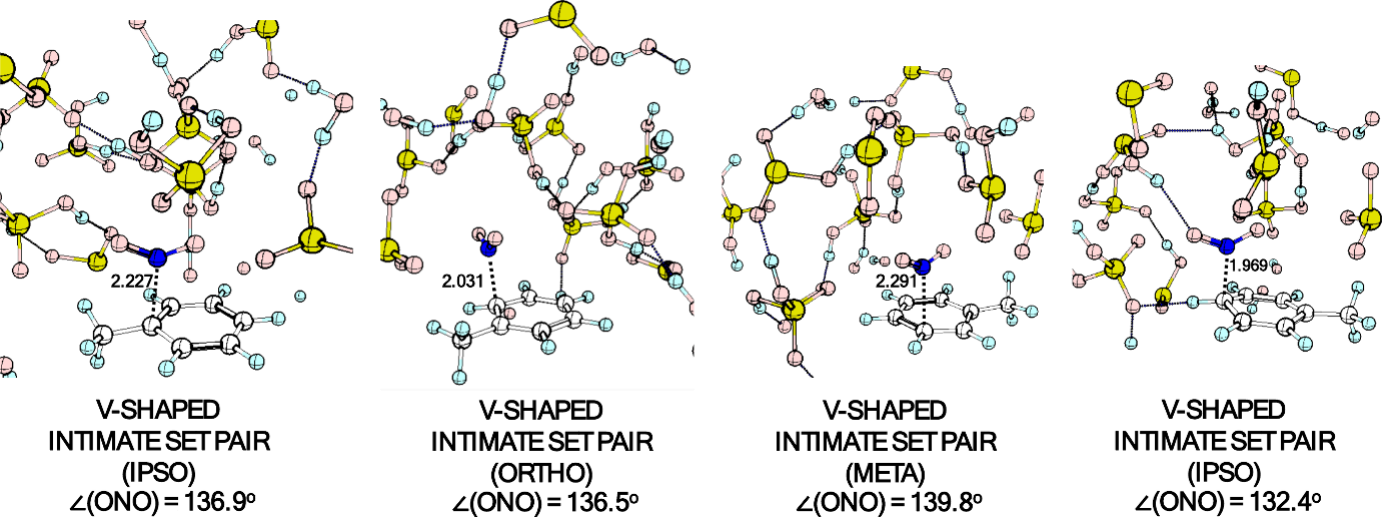
Optimized geometries of the V-shaped intimate SET pairs at several positions of the toluene ring.

As an example, after 7.5 ps for the beginning of one of the trajectories, the formation of a σ-bond between the NO_2_ group and toluene was observed, affording a σ-complex at the *para* position (Figure 7f). Subsequently, at 8.5 ps, after ∼ 1 ps from the formation of the σ-complex, a conjugate base abstracts a proton from the carbon bonded to the NO_2_ group, yielding the product *p*-nitrotoluene. Shortly after, one of the nitro group’s oxygen atoms was protonated, forming the final stable and deactivated species, protonated *p*-nitrotoluene, as shown in Figures 7g and 7h. Again, no hydrogen bonding between the acid medium and the NO_2_^+^ intermediate was observed.

Geometry optimization of the frames identified as σ–complexes affords the structures shown in Figure 9.

**Figure 9:**
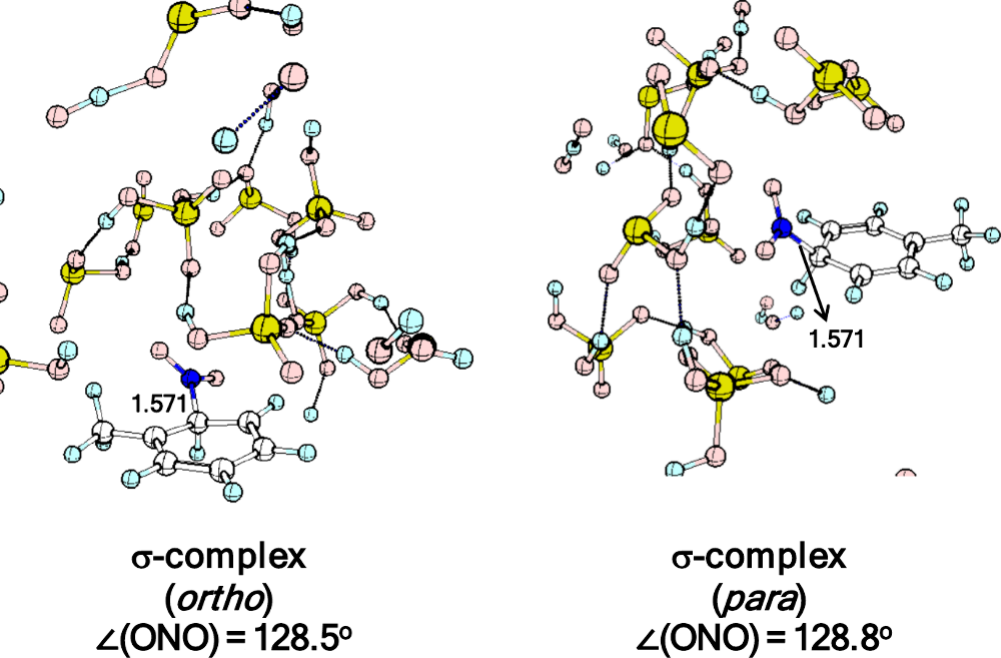
Optimized geometries of the σ-complexes at ortho and para positions.

Electronic property calculations, including HOMO, LUMO, and spin density analyses, as well as Bader charge analysis, were performed for the stages involving the interaction of the nitronium ion with the conjugate base (NO_2_^+^·HSO_4_^–^), the formation of the intimate SET complex, the σ-complex formation, and the generation of the *p*-toluene product, as shown in Figure 10. Calculations of such electronic properties employing the PBE and PBE0 functionals were performed to evaluate the potential influence of electron self-interaction errors inherent to the pure GGA PBE functional, which may lead to charge delocalization in charge-transfer processes and thus affect the accurate description of charge-separated states. The comparison between the functionals revealed consistent qualitative results, as detailed in the Supporting Information.

**Figure 10:**
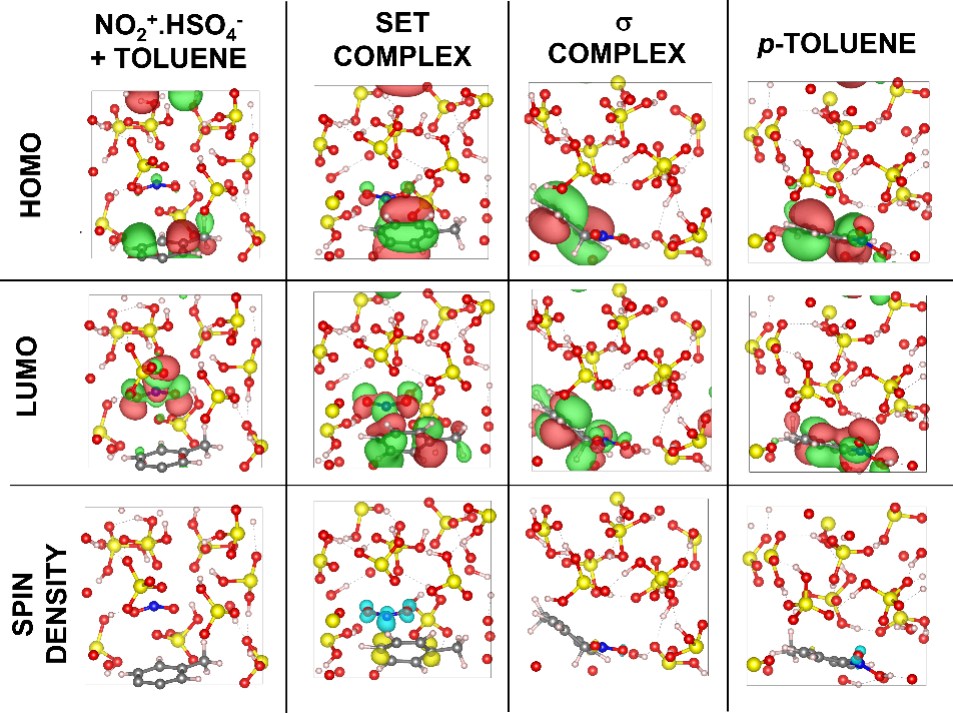
HOMO and LUMO orbitals and spin density maps for the key stages of the toluene nitration mechanism at the *para* position at 300 K, computed using the PBE0 functional. Atom color scheme: red = oxygen, yellow = sulfur, blue = nitrogen, gray = carbon, white = hydrogen. For HOMO and LUMO representations, red indicates positive density and green indicates negative density. For spin density maps, yellow represents positive spin density and blue represents negative spin density. An isosurface level of 0.025 was used for the HOMO and LUMO orbitals and 5 × 10^–9^ for the spin density in all cases.

The HOMO and LUMO orbitals reveal that, in the initial stage, the nitronium ion primarily interacts with the surrounding sulfuric acid species, forming the NO_2_^+^·HSO_4_^–^ adduct. As the reaction progresses, the ion begins to interact with toluene, leading to the formation of the intimate SET complex. In this configuration, constructive overlap between the positive orbital lobes of the nitronium ion and the *para*-carbon of toluene indicates the onset of bonding interactions.

As shown by the spin density corresponding to the SET complex in Figure 10, unpaired electron density appears on the nitronium ion, characteristic of a radical intermediate associated with a single-electron transfer (SET) process. Concurrently, spin density emerges on toluene, mainly at the ipso and para positions, consistent with mechanistic proposals reported in previous studies.^13^

The subsequent formation of the σ-complex is characterized by the localization of the HOMO and LUMO within the newly formed covalent framework. Following the deprotonation and protonation steps, the protonated *p*-nitro-toluene product is generated. In this final stage, the frontier orbitals remain localized with minimal interaction with the surrounding medium, indicating product stabilization and electronic deactivation. Moreover, after the formation of the σ-complex and the p-toluene product, no spin density is observed, consistent with electron pairing and the conclusion of the radical process.

Bader partial charge values for the key stages of the nitration reaction are summarized in Table 1. In the initial stage, corresponding to the interaction of the nitronium ion with its conjugate base (NO_2_^+^·HSO_4_^–^), the nitronium ion exhibits a partial charge of +0.52*e*, indicating stabilization by the HSO_4_^–^ anion. During the formation of the intimate SET complex, the nitronium ion undergoes a reduction in positive charge, while toluene gains electron density, supporting the occurrence of a single-electron transfer event without the establishment of a covalent bond.

**Table 1:** Partial Charges of the Nitronium Ion and Toluene at the Main Stages of the Toluene Nitration Mechanism: Interaction with the Conjugate Base (NO_2_^+^·HSO_4_^–^), Formation of the Intimate SET Complex, Formation of the σ-Complex, and Generation of the Protonated *p*-Nitrotoluene Product^a^

Para Position Attack				
Reactant	NO_2_^+^·HSO_4_^–^ + Toluene	SET Complex	σ-Complex	*p*-nitrotoluene
q(NO_2_)	+0.62	–0.30	–0.51	+1.03
q(Toluene)	+0.12	+1.22	+1.46	

Ortho Position Attack				
Reactant	NO_**2**_^+^·HSO_4_^–^ + Toluene	SET Complex	σ-Complex	*o*-nitrotoluene
q(NO_2_)	+0.39	+0.43	–0.47	+0.96
q(Toluene)	+0.11	+1.07	+1.64	

a The unit of charge is *e*, the modulus of the electron charge (*e* = 1.60217663 × 10^–19^ coulombs). All calculations were performed using the PBE0 functional.

In the subsequent σ-complex stage, the nitro group, strongly electron-withdrawing, extracts electron density from toluene through the newly formed σ-bond, resulting in a combined charge of approximately +0.90*e*. Finally, in the protonated *p*-toluene product, the total charge increases again to +0.99*e*, consistent with charge redistribution and electronic stabilization after completion of the reaction.

#### Trajectories Leading to the Attack at the Ortho Position

Another set of trajectories, starting from different initial configurations, afforded the nitration product at the ortho position. In general, these trajectories exhibit trends similar to those leading to nitration at the para position, that is, an SET step resulting in the relatively long-lived V-shaped intimate SET complex, which subsequently evolves into the σ-complex, as shown in Figure 11. This intermediate, after deprotonation, yields the final product, *o*-nitrotoluene.

**Figure 11:**
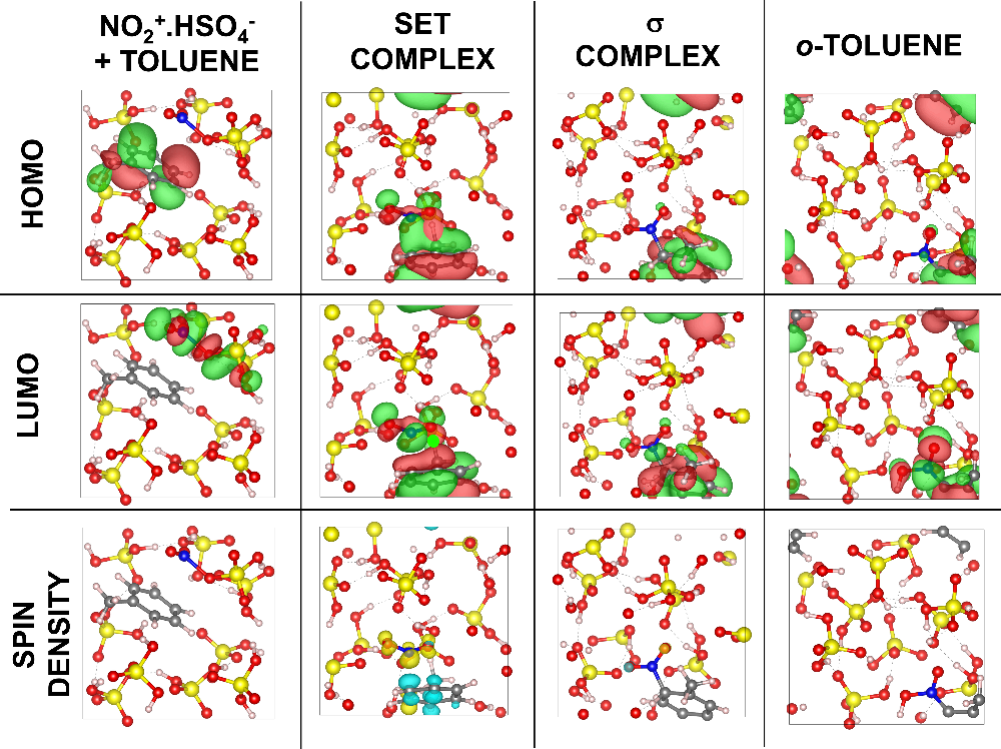
HOMO and LUMO orbitals and spin density maps for the key stages of the toluene nitration mechanism at the *ortho* position at 300 K, computed using the PBE0 functional. Atom color scheme: red = oxygen, yellow = sulfur, blue = nitrogen, gray = carbon, white = hydrogen. For HOMO and LUMO representations, red indicates positive density and green indicates negative density. For spin density maps, yellow represents positive spin density and blue represents negative spin density. An isosurface level of 0.025 was used for the HOMO and LUMO orbitals and 5 × 10^–9^ for the spin density in all cases.

As shown in Figure 11, the HOMO, LUMO, and spin density maps for *o*-nitrotoluene display patterns analogous to those observed for *p*-nitrotoluene, confirming that both reaction channels proceed through comparable single-electron transfer and σ-complex formation steps. The appearance of spin density in the SET complex further corroborates the occurrence of the single-electron transfer process prior to covalent bond formation.

For the *ortho*-nitration pathway, the Bader partial charge analysis (Table 1) shows that the nitronium ion initially carries a charge of +0.39 *e*, indicating stabilization by the HSO_4_^–^ anion. Upon formation of the intimate SET complex, its charge slightly increases to +0.43 *e*, while toluene becomes more positively charged (+1.07 *e*), consistent with electron transfer without covalent bond formation. In the σ-complex, the nitro group becomes negatively charged (−0.47 *e*) as it withdraws electron density from toluene, whose charge rises to +1.64 *e*. In the final *o*-nitrotoluene product, the nitro group regains a positive charge of +0.96 *e*, reflecting charge redistribution and stabilization similar to that observed in the *para*-nitration pathway.

#### Attack at the Meta and Ipso Positions

No trajectories lead to the nitrated products at either *meta* or *ipso* positions, although the corresponding SET intimate pair intermediates at these positions were found. Interestingly, as mentioned previously, the calculations show that the intimate SET pair intermediate at a given position may explore all the available positions of the aromatic ring. Their persistence is observed especially at the *ipso* and *para* positions, coincidentally where the higher HOMO coefficients are in the toluene. The intimate SET pair at *ipso* position often evolves to the one at the *ortho* position, which eventually can evolve to the σ-complex at such a position, which affords the *ortho*-substituted nitrated products after deprotonation. This may indicate that the *ortho* nitration can be the outcome of the attack at both *ortho* and the *ipso* position of the aromatic ring, considering that the *ipso* attack is favored by the high HOMO coefficient at this position. This feature would help to explain why the *ipso* attack is observed many times whenever good leaving substituents are present.^34–38^

A feature observed in all trajectories that formed the V-shaped intimate SET pair is that this intermediate is somehow long-lived, and a given intermediate formed at a certain position of the aromatic ring can explore the other positions. Their decreasing relative stability is *ipso* > *para* > *ortho* > *meta*. In all trajectories, only the V-shaped intimate SET pair complexes that evolved to the σ-complexes (arenium ions) were the ones where the NO_2_ moiety is at *ortho* and *para* positions.

Their relative energy averages can be used to evaluate their relative proportion. From the different trajectories, the average energy of the frames of each species was used to determine the relative energies among them. By doing this, the relative energies for the ortho/meta/para V-shaped intimate SET pair are respectively 0.7/3.4/0.0 kcal/mol. If this complex is the key to the formation of the position selectivity in the nitration of toluene, proportions among them should express the relative quantities of the nitrated products at each position. Experimentally, nitration of toluene in sulfonitric solutions gives nitrated products in about 57% yield at the *ortho* position, 5% at the *meta* position, and 38% at the *para* position. Considering that toluene has two *ortho*, two *meta*, and one *para* positions available for nitration and that the intimate SET pair is responsible for positional selectivity, computed ratios from considering the SET intimate pair would afford ratios of 39.5% *ortho*: 0.4% *meta*: 60.1% *para*, expressing the preference for the *ortho* and *para* positions, although with an inversion between *ortho* and *para* in relation to the observed experimentally. It would also be possible that part of the ortho product could come from the ipso SET intimate pair, which is the most stable of these complexes (by ∼ 3 kcal/mol) but leads to no nitrated product. On the other hand, energies of the σ-complexes do not correlate with the positional selectivity at all. Thus, these results indicate that the positional selectivity could be defined by the stabilities of the intimate SET pairs.

### Other Reacting Trajectories: Oxygen Transfer

Other reaction outcomes leading to oxygenated products were also observed. The detailed analysis of these reaction pathways can be found in the Supporting Information. In summary, after the SET step, a Λ-shaped [NO_2_·ArH]^+^ SET intimate complex is formed, with two main conformational variations: one with a hydrogen bond to the aromatic ring and the other without this feature. These complexes have an average lifetime of 20 ps and eventually collapse into different oxygenated products, which can tautomerize into one another.

#### Outlook

Scheme 2 summarizes the results of all BOMD calculations carried out. Interestingly, no hydrogen bonding between the nitronium ion (NO_2_^+^) or the NO_2_ radical formed after the SET step is protosolvated, i.e., forms hydrogen bonding with the acid solvent. Acid molecules prefer in general to make an extended hydrogen-bonded net, in which the reacting species are embedded. In some cases, protonation may take place (*e.g*,, to protonation of the nitrated product), but in general, no specific interaction takes place.

**Scheme 2:**
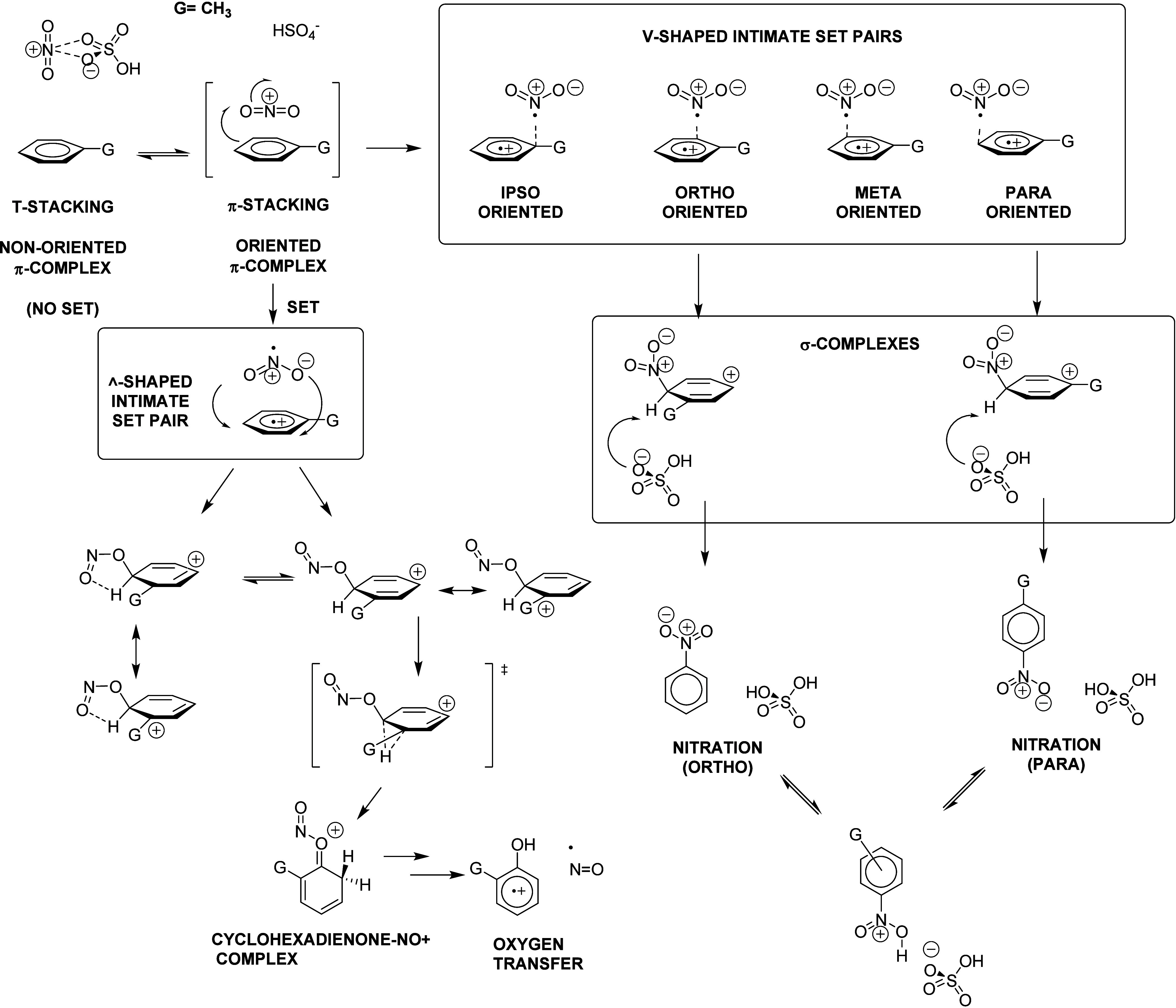
Reaction Pathways Observed from the Toluene Reaction in a Sulfonitric Mixture Observed in BOMD Simulations: Nitration, Cyclohexadienone–NO Complex Formation, and Oxygen Transfer

## Conclusions

Concluding, one can say that the data confirms previous proposals that the nitronium cation, NO_2_^+^, is being formed in the sulfonitric mixture, but through diprotonation of the HNO_3_ by H_2_SO_4_. The nitration of toluene occurs through a SET process, either in polar protic solvents, here represented by the sulfonitric mixture, or aprotic solvents. This contribution shows many different reaction outcomes for the reaction, mainly about the SET just take place in case of the nitronium ion is parallel to the pi-system, and within the same solvent cage. Depending on how the vibration of the NO_2_^+^ takes place, one can have a V-shaped [NO_2_.ArH]^+^ complex, which leads to the intimate SET pair, which is more persistent than the Λ-shaped [NO_2_.ArH]^+^ complex. The intimate SET pair is preferentially formed at the *ipso*, *para* and *ortho* position, but also occurs at the meta position. They have a lifetime long enough to explore the different sites of the aromatic ring. Selectivity is easily explained by the stability of the V-shaped intimate SET pair rather than the σ-complexes. The V-shaped The intimate SET pair [NO_2_.ArH]^+^ complexes at the ortho and para positions were the only ones that evolve to their respective σ-complexes, which, after being deprotonated, afford the nitrated product, eventually further O-protonated by the acid medium. The Λ-shaped intimate SET pair [NO_2_.ArH]^+^ complex quickly evolves to oxygen transfer to the aromatic ring. Depending on its orientation, due to internal hydrogen bond formation, it can rearrange to the phenol or stay as this O-alkylated complex. There is no evidence for the BOMD calculations that superelectrophiles or protossolvation is taking place in this system.

## Supplementary Material



## Data Availability

All the original data from the simulations, including input and output files, as well as videos of selected trajectories, can be found at the Zenodo repository at 10.5281/zenodo.15722757.

## Notes

Comparison between PBE and PBE0 of HOMO, LUMO, spin density, and Bader charges for selected processes and results and discussion about the oxygen transfer processes (PDF)
