# Supplementary material for: Nitration Mechanism of Aromatics: Lessons from Born–Oppenheimer Molecular Dynamics
Source: ACS Phys Chem Au. 2025 Nov 17;6(1):103–13. doi: 10.1021/acsphyschemau.5c00086 (PMC12856663; doi:10.1021/acsphyschemau.5c00086)
Supplement: Supplementary file 1 [file pg5c00086_si_001.pdf]

Supporting Information

for

The Nitration Mechanism of Aromatics: Lessons from Born-Oppenheimer Molecular Dynamics

*Fabio J. F. S. Henrique and Pierre M. Esteves**

Instituto de Química, Universidade Federal do Rio de Janeiro, Av. Athos da, Silveira Ramos, 149, CT, A-622, Cid. Univ., Rio de Janeiro, 21941-909, RJ, Brazil.; pesteves@iq.ufrj.br

KEYWORDS. Nitration, electrophilic, aromatic substitution, Born-Oppenheimer Molecular Dynamics, superelectrophile.

Comparison between PBE and PBE0 for toluene nitration ---------------------------------*S2*

Oxygen Transfer Reactions---*S3*

Comparison between PBE and PBE0 for toluene nitration

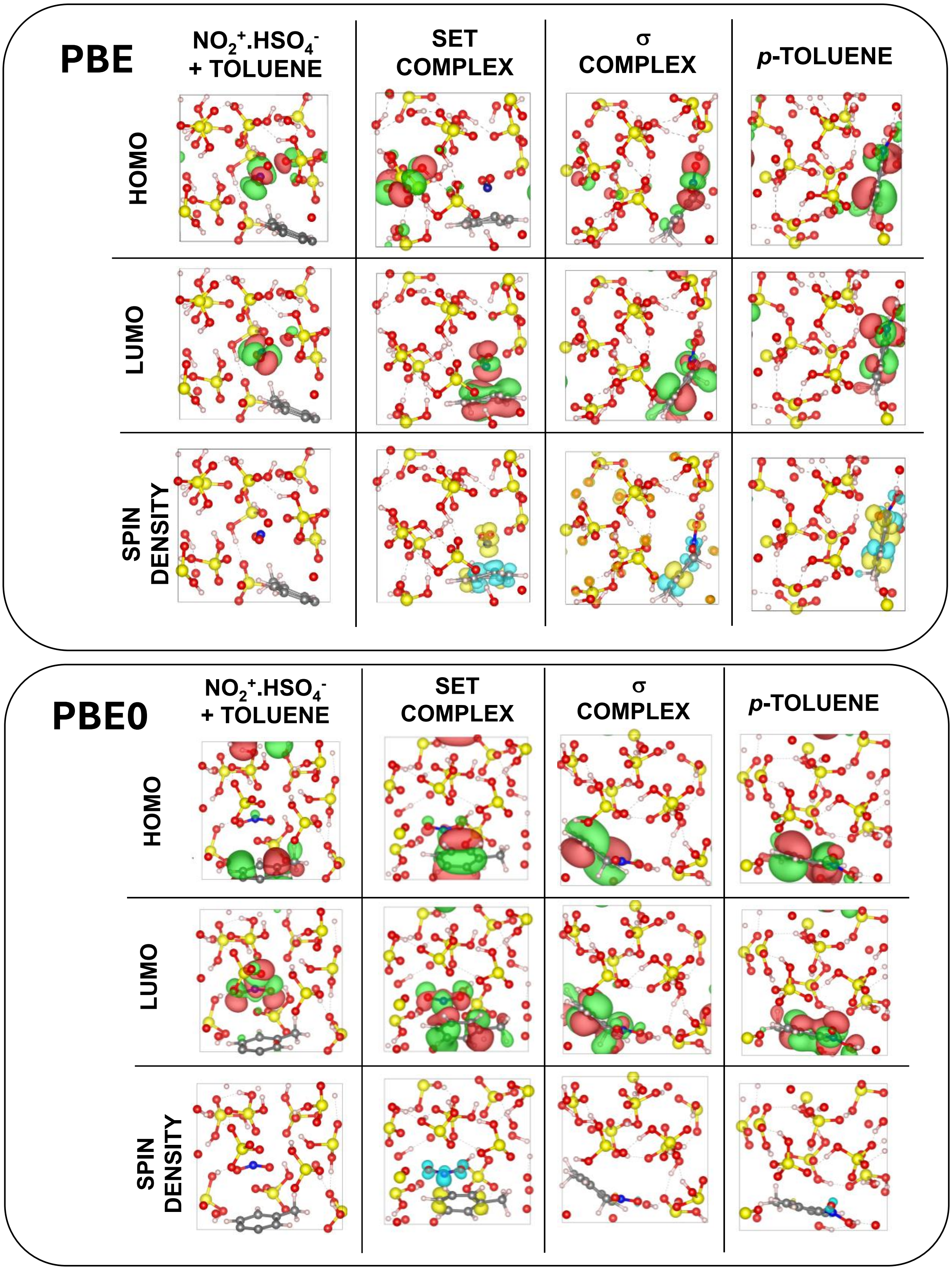

Figure S1. HOMO and LUMO orbitals and spin density maps for the key stages of the toluene nitration mechanism at 300 K, calculated with the PBE functional. Atom color

scheme: red = oxygen, yellow = sulfur, blue = nitrogen, gray = carbon, white = hydrogen. For HOMO and LUMO representations, red indicates positive density and green indicates negative density. For spin density maps, yellow represents positive spin density and blue represents negative spin density. An isosurface level of 0.025 was used for the HOMO and LUMO orbitals, and 5×10^{-9} for the spin density in all cases.

Table S1. Partial charges of the nitronium ion and toluene at the main stages of the toluene nitration mechanism, calculated with the PBE and PBE0 functionals: interaction with the conjugate base ($NO_2^+ \cdot HSO_4^-$), formation of the intimate SET complex, formation of the σ-complex, and generation of the protonated *p*-nitrotoluene product. The unit of charge is *e*, the modulus of the electron charge ($e = 1.60217663 \times 10^{-19}$ coulombs).

Para position atack (PBE)	Reactant	$NO_2^+ \cdot HSO_4^-$ + Toluene	SET Complex	σ-Complex	*p*-nitrotoluene
	q(NO_2)	+0.52	0.00	-0.36	+0.99
	q(Toluene)	+0.10	+0.91	+1.26	
**Para position atack (PBE0)**	**Reactant**	**$NO_2^+ \cdot HSO_4^-$ + Toluene**	**SET Complex**	**σ-Complex**	***p*-nitrotoluene**
	q(NO_2)	+0.62	-0.30	-0.51	+1.03
	q(Toluene)	+0.12	+1.22	+1.46	

Oxygen Transfer Reactions

Some simulations lead to alternative minor products, such as oxygen transfer to the aromatic ring. In these simulations, the reaction of the nitronium ion with toluene was observed at the *ortho* position, resulting in the formation of *ortho*-cresol and nitric oxide

(NO). Figure S2 shows the evolution of the ONO bond angle and the distance between the nitronium group and the *ortho*-carbon of toluene throughout the simulation.

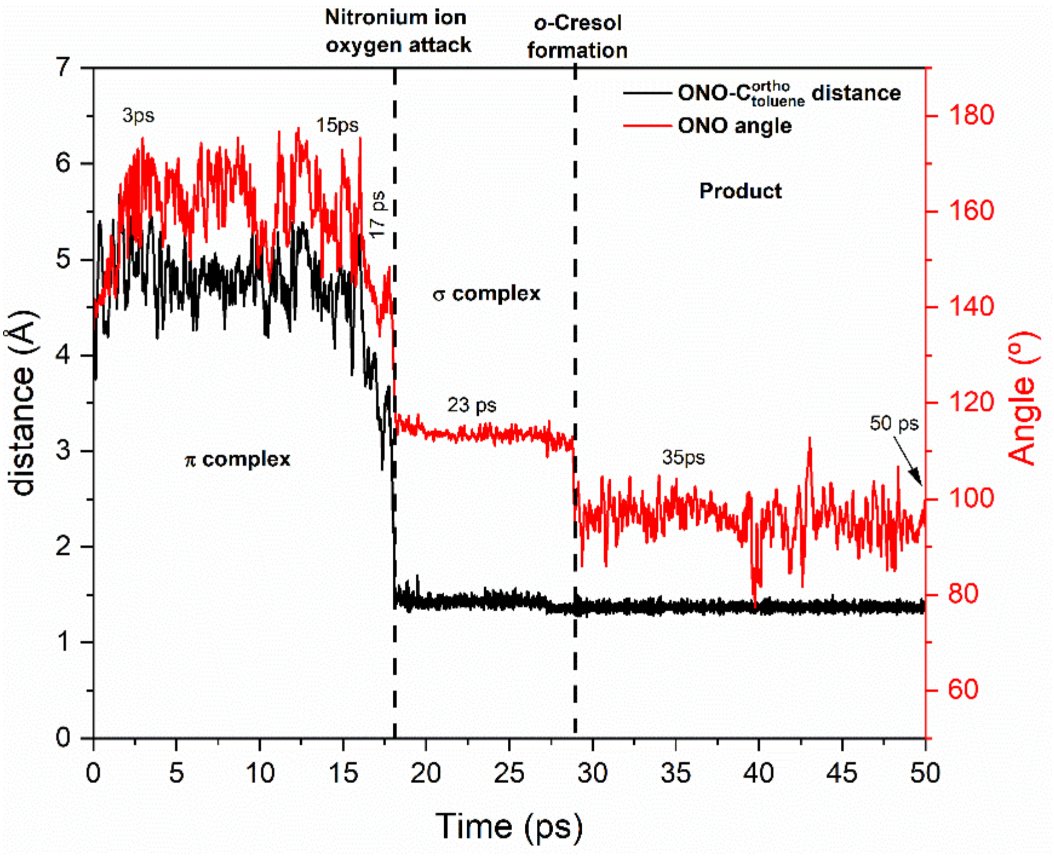

Figure S2. Time evolution of the distance between the NO_2 group and the para-carbon atom of toluene, and the ONO bond angle of the nitronium ion during the o-cresol process formation, at 300 K.

At the beginning of the reaction, the nitronium ion remained stabilized by the conjugate base HSO_4^-, as seen in previous nitration simulations. However, around 2 ps, the nitronium ion began interacting with toluene and formed a stable π-complex, as shown in Figures S3a and S3b, which persisted for approximately 15 ps with an average ONO angle of ~160°. At 17 ps (Figure S3c), the π-complex dissociated, and the nitronium ion adopted a bent geometry with an ONO angle of 136°, similar to what was previously observed following a single electron transfer (SET) event in the para-nitration simulation,

but with the NO_2 moiety assuming a ∧-shaped form in the $[NO_2.ArH]^+$ complex. Then, at 18 ps, one of the oxygen atoms of the NO_2 moiety attacked the *ortho*-carbon of toluene, forming a σ-complex (Figure 11d). This σ-complex remained stable for about 11 ps. At 29 ps, deprotonation of the attacked carbon occurred, forming 2-methylphenolate, which was rapidly protonated by sulfuric acid, yielding *ortho*-cresol and a NO group. These products remained stable for more than 20 ps, and Figures S3e and S3f show that the NO group stayed near the *ortho*-cresol for the remainder of the simulation.

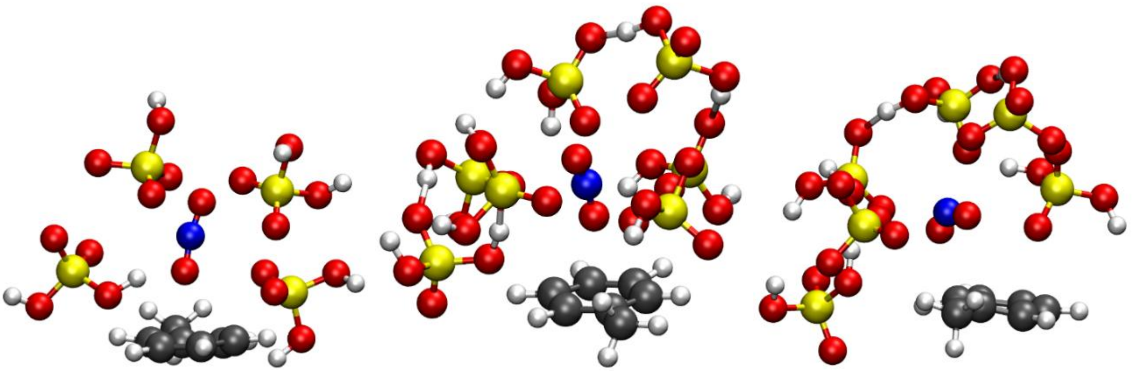

(a) (b) (c)

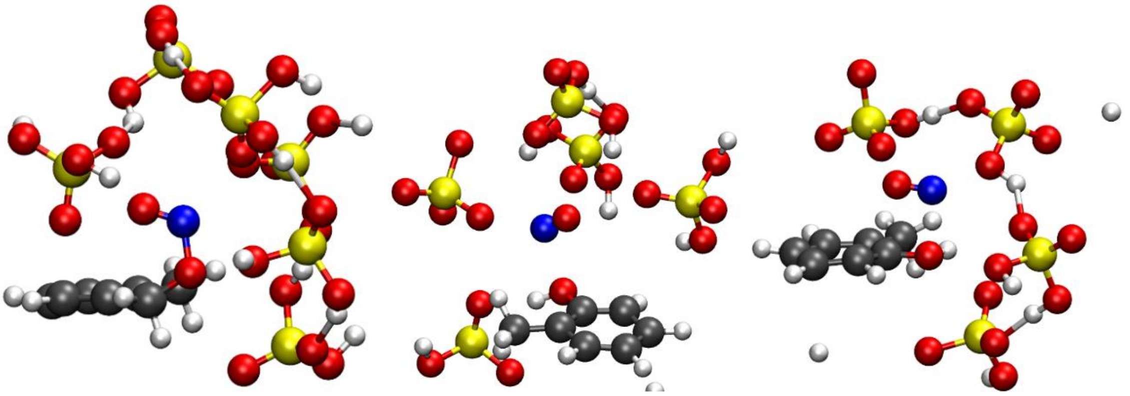

(d) (e) (f)

Figure S3. Solvation sphere with a 4 Å radius around the nitronium ion at selected time frames from the *o*-cresol formation simulation at 300 K: (a) 3 ps, (b) 15 ps, (c) 17 ps, (d)

23 ps, (e) 35 ps, and (f) 50 ps. Atom color scheme: red = oxygen, yellow = sulfur, blue = nitrogen, gray = carbon, white = hydrogen.

It is noteworthy that the V-shaped [NO_2.ArH]$^+$ complex afforded the nitration product, while the ʌ-shaped [NO_2.ArH]$^+$ complex led to oxygen transfer to the aromatic ring (Scheme S1). This might be related to the electrostatic attraction between the negatively charged oxygen atoms in NO_2 and the positively charged aromatic ring, which are closer in ʌ-shaped [NO_2.ArH]$^+$ SET intimate complex than in the V-shaped complex.

Scheme S1. Reaction pathways from the [NO_2·ArH]$^+$ SET intimate complexes: (a) nitration via the V-shaped complex, and (b) oxygen transfer via the ʌ-shaped complex.

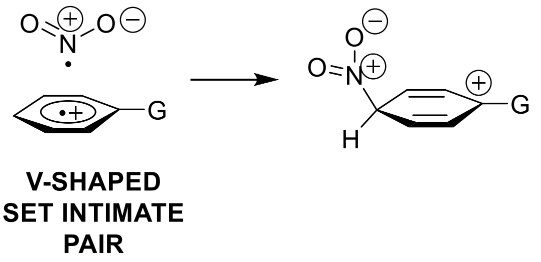

(a)

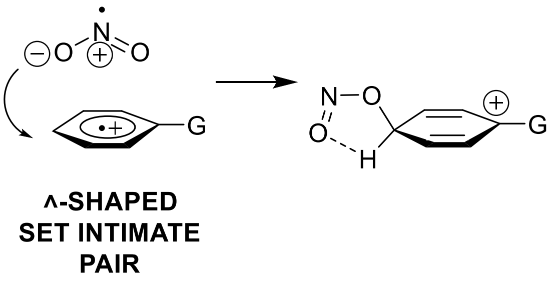

(b)

Electronic property calculations, including HOMO, LUMO, and spin density analyses, as well as Bader charge analysis, were performed for the selected frames of the

o-cresol formation simulation, shown in Figure S4. The HOMO and LUMO orbitals at 3 and 15 ps (π-complex frames) reveal strong interactions between the nitronium ion and toluene, especially in the HOMO. At 17 ps, when the nitronium ion became bent (~136°), changes in the HOMO of toluene were observed, with π-character orbitals now localized on the *ortho* and *para* carbons. Additionally, spin density on the nitronium ion was predominantly positive, while the *ipso* and *para* carbons of toluene also exhibited slightly positive spin density, indicating the occurrence of SET and the formation of two radical species.

The oxygen attack by the nitronium ion at the *ortho*-position is depicted at 23 ps and confirms that the attack occurred from the anionic character of the oxygen atoms of the neutral NO_2 moiety. The HOMO, LUMO, and spin density distributions suggest a stable intermediate. In the product frames (35 and 50 ps), the HOMO becomes localized on the NO species, while the LUMO is distributed across both the *o*-cresol and NO molecules. Spin density results, especially at 50 ps, show the presence of unpaired electrons in both the NO and *o*-cresol molecules, particularly at the *ortho* carbon atoms, suggesting the persistence of radical character.

Frame	SYSTEM	HOMO	LUMO	SPIN DENSITY
3 ps	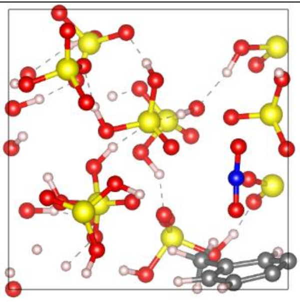	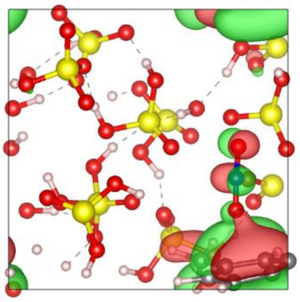	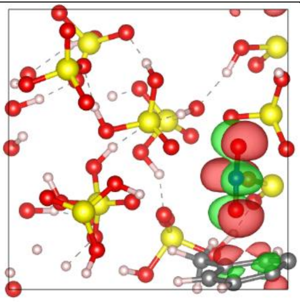	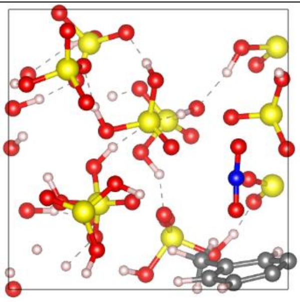

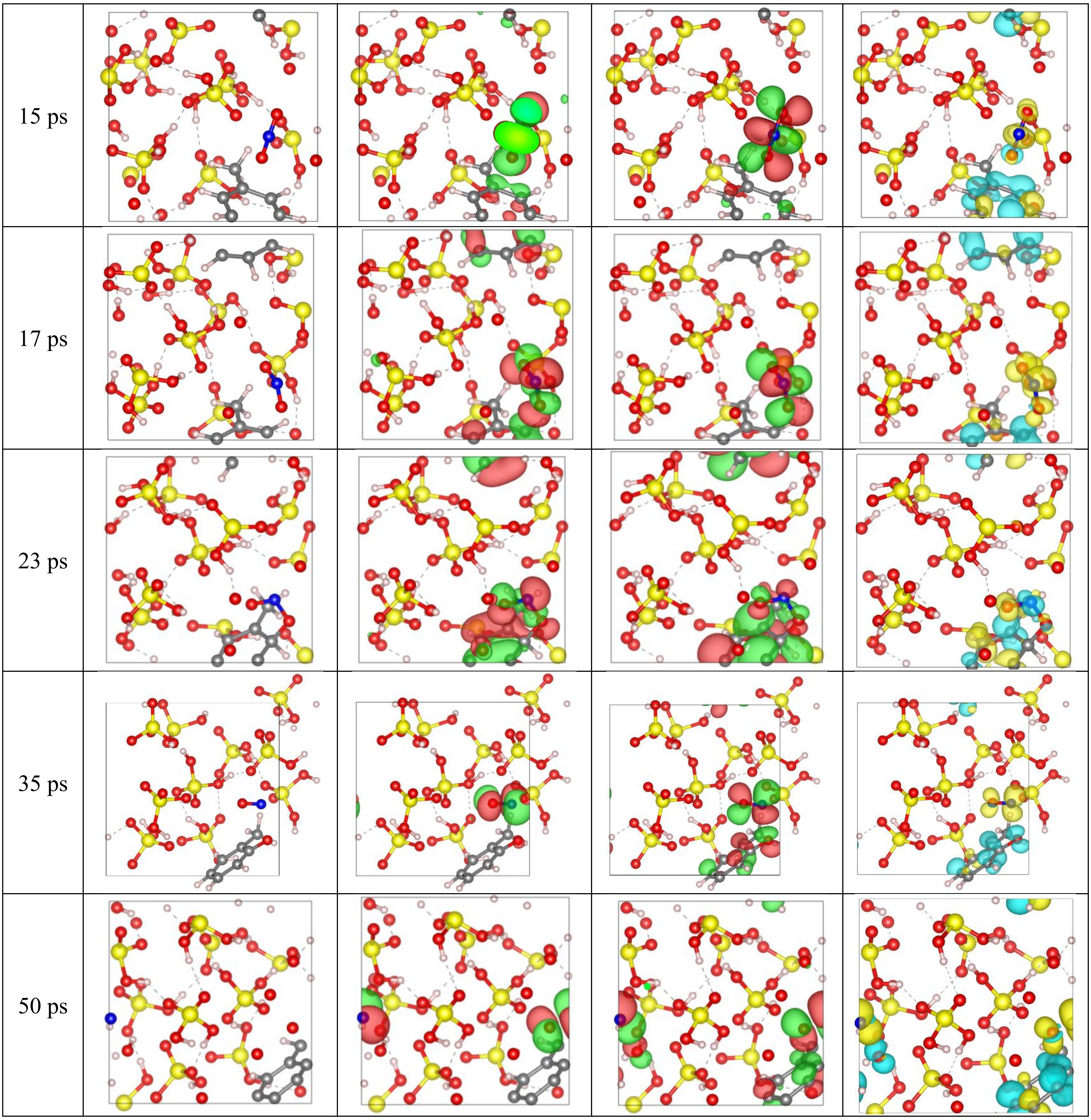

Figure S4. HOMO and LUMO orbitals, and spin density maps at representative time frames from the *o*-cresol formation simulation at 300 K. Atom color scheme: red = oxygen, yellow = sulfur, blue = nitrogen, gray = carbon, white = hydrogen. For HOMO and LUMO maps, red indicates positive orbital density and green indicates negative

density. For spin density maps, yellow represents positive spin density and blue indicates negative spin density. An isosurface value of 0.025 was used for HOMO and LUMO, and 5×10^{-9} for spin density in all cases. All calculations were performed using the PBE functional and the cc-pVTZ basis set.

The Bader partial charges calculated for the selected frames of the *o*-cresol formation simulation are presented in Table S2. At 3 and 15 ps, the nitronium ion slightly withdrew electronic charge from toluene, rendering it mildly positively charged compared to the initial stages of the *para*-nitration simulation (see Table 1). At 17 ps, the partial charges (+0.45*e* for NO_2 and +0.46*e* for toluene), along with the spin density images from Figure S4, confirm the SET event.

At 23 ps, corresponding to the σ-complex, the nitro group showed a partial charge of –0.66*e* while toluene exhibited a significant positive charge of +1.58*e*, suggesting that the electron-withdrawing effect of NO_2 was enhanced due to the oxygen-mediated attack. Finally, the partial charges of the NO and o-cresol products at 35 and 50 ps were approximately +0.50*e* for both species, reinforcing the presence of radical character in both molecules.

Table S2. Partial charges of the nitronium ion and toluene at selected time frames (3 ps, 15 ps, 17 ps, and 23 ps) during the *o*-cresol formation simulation. Also shown are the partial charges of the final products, *o*-cresol and NO, at 35 ps and 50 ps. Unity of charge is *e*, the modulus of the electron charge (e = 1.60217663 × 10^{-19} coulombs). All calculations were performed using the PBE functional and the cc-pVTZ basis set.

Reactant	3 ps	15 ps	17 ps	23 ps	Product	35 ps	50 ps
**q(NO_2)**	+0.66	+0.71	+0.45	-0.66	**q(NO)**	+0.47	+0.49
**q(Toluene)**	+0.28	+0.23	+0.46	+1.58	**q(*o*-cresol)**	+0.54	+0.52

Reacting trajectories: cyclohexadienone-NO complex/cresol radical cation-NO formation

The fourth simulation involving the sulfonitric mixture and toluene was carried out for 40 ns. In this simulation, the reaction of the nitronium ion with toluene was observed at the *ortho* position, resulting in the formation of 2-methylcyclohexa-2,4-dien-1-one and nitric oxide (NO). Figure S5 shows the evolution of the ONO bond angle and the distance between the nitronium group and the *ortho*-carbon of toluene throughout the simulation.

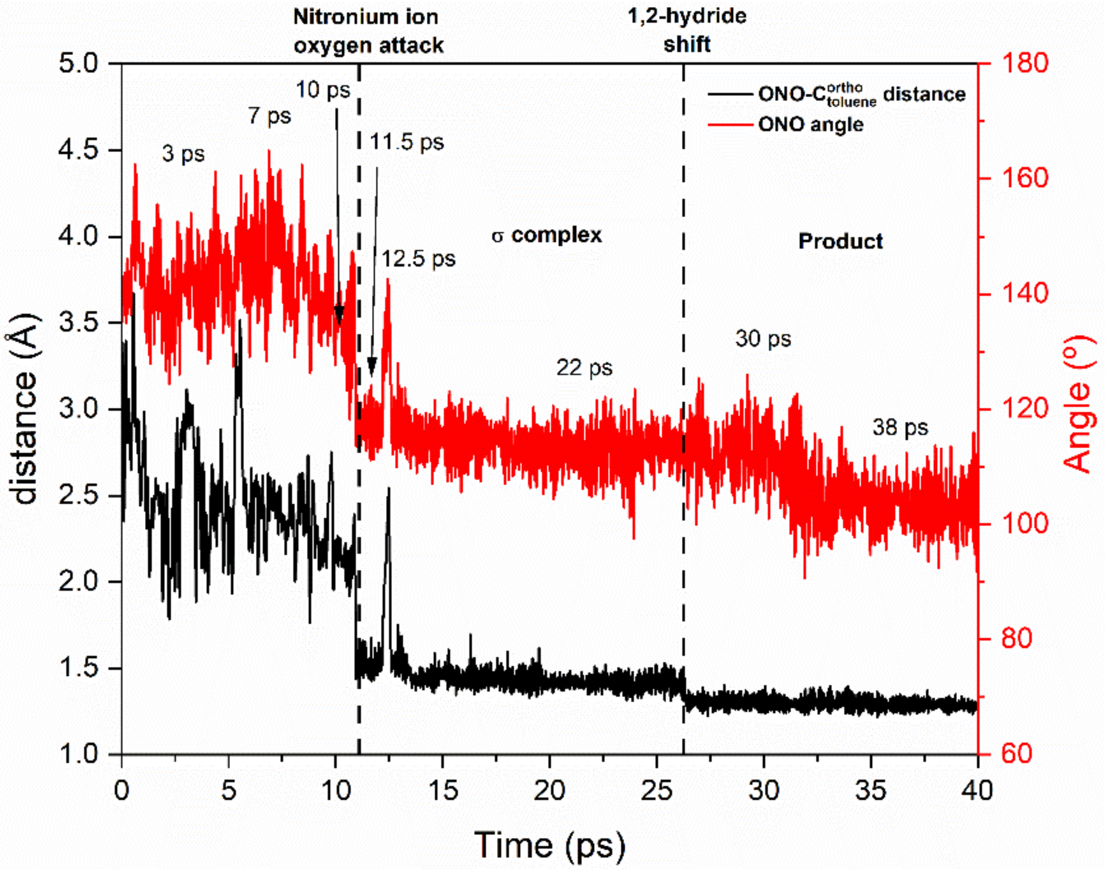

Figure S5. Time evolution of the distance between the NO_2 group and the para-carbon atom of toluene, and the ONO bond angle of the nitronium ion during the cyclohexadienone-NO complex formation, at 300 K.

At the beginning of the reaction, the nitronium ion remained stabilized by the conjugate base HSO_4^-. For the first 10.8 ps, it interacted continuously with both HSO_4^- and toluene, maintaining an average ONO bond angle of approximately 142°, occasionally reaching 136°, a value previously associated with single-electron transfer (SET) events (Figures S6a, S6b and S6c). However, the intermediate remained stabilized in a ʌ-shaped conformation, which did not favor a nucleophilic attack by the radical character localized on the nitrogen atom. After 10.8 ps, this ʌ-shaped conformation enabled the oxygen atom of the nitronium ion to attack the ortho-carbon of toluene,

forming a ∧-shaped $[NO_2{\cdot}ArH]^+$ SET intimate complex, as shown in Scheme 2 and Figure S6d. At 12.5 ps, the nitronium ion was observed to detach from the ortho-carbon of toluene, accompanied by an increase in the ONO bond angle from ~120° to ~140° (Figure S6e). However, shortly thereafter, the NO_2^+ ion reapproached and reformed the ∧-shaped $[NO_2{\cdot}ArH]^+$ SET intimate complex, suggesting that this step is likely reversible. This intermediate remained stable for approximately 11 ps (Figure S6f). In this ∧-shaped $[NO_2{\cdot}ArH]^+$ SET complex, the oxygen atom formed a strong hydrogen bond with the hydrogen atom bonded to the ortho-carbon of toluene. This interaction hindered the removal of that proton by a conjugate base, a step that was otherwise observed in the o-cresol formation reaction. After 26 ps of simulation, the sterically hindered hydrogen underwent a 1,2-hydride shift to the meta-position of toluene, resulting in the formation of a complex between 2-methylcyclohexa-2,4-dien-1-one and NO^+. These two moieties remained stable for over 14 ps, with the NO group staying close to the carbonyl moiety of the methylcyclohexadienone molecule, as illustrated in Figures S6f and S6h.

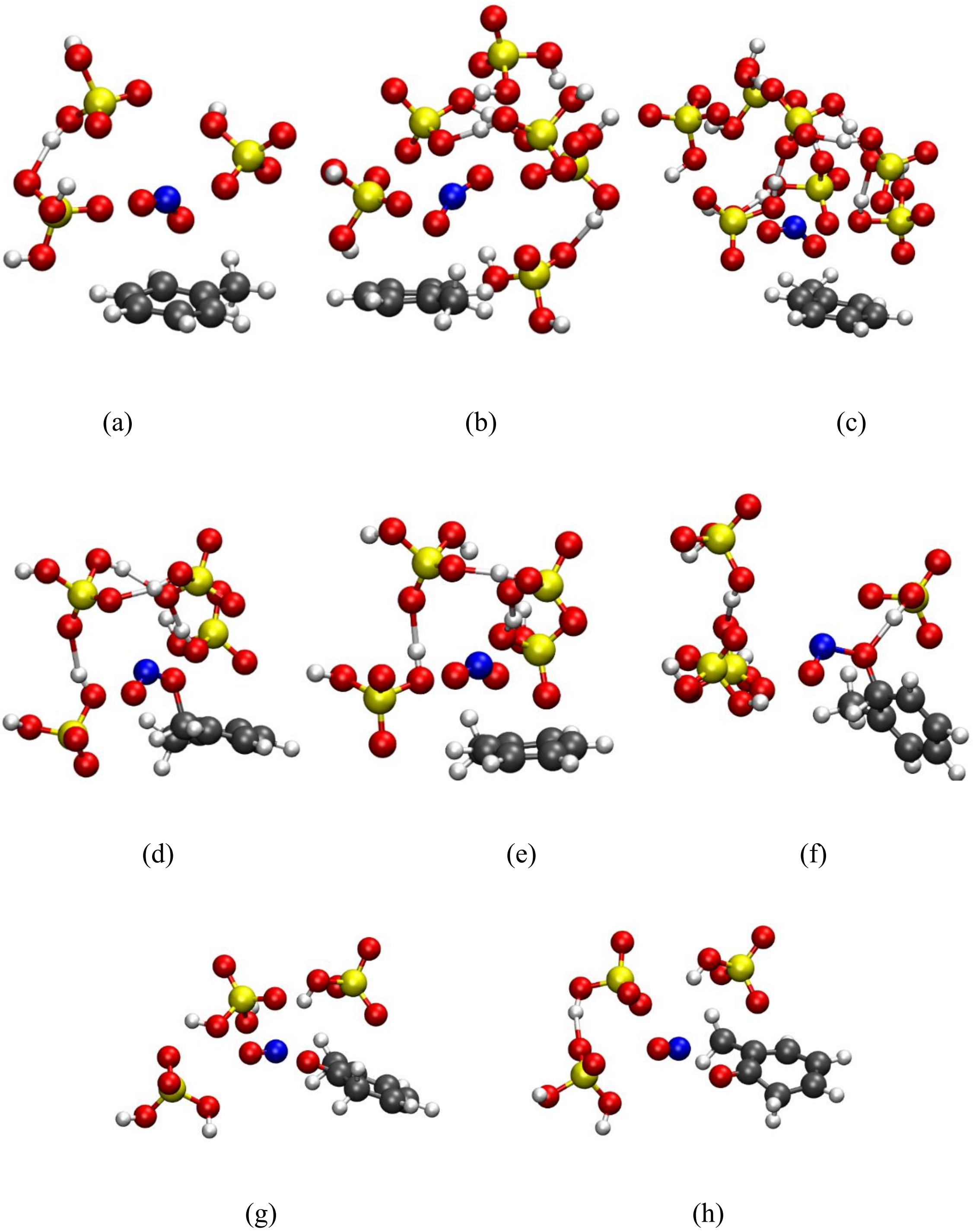

Figure S6. Solvation sphere with a 4 Å radius around the nitronium ion at selected time frames from cyclohexadienone-NO complex formation simulation at 300 K: (a) 2 ps, (b) 7 ps, (c) 10 ps, (d) 11.5 ps, (e) 12.5, (f) 22 ps, (g) 30 ps and (f) 38 ps. Atom color scheme: red = oxygen, yellow = sulfur, blue = nitrogen, gray = carbon, white = hydrogen.

Electronic property calculations, including HOMO, LUMO, and spin density analyses, along with Bader charge analysis, were conducted for selected frames of the methylcyclohexadienone–NO complex formation simulation, as illustrated in Figure S7. The HOMO and LUMO orbitals at 2 and 7 ps, corresponding to the interaction between the nitronium ion and the surrounding environment, revealed strong electronic interactions between the nitronium ion and toluene. These interactions were especially pronounced in the HOMO, while the LUMO was predominantly localized on the nitronium ion, suggesting its electrophilic character. Additionally, spin density analysis at 7 and 10 ps revealed regions of positive spin density (highlighted in yellow) both on the nitronium ion and on the ortho and opposite meta positions of toluene, indicating that a single electron transfer (SET) event may have occurred between the interacting species.

At 11.55 ps, as also observed in the reaction pathway leading to *o*-cresol formation, the electrophilic attack was initiated via the oxygen atom of the nitronium ion, attributed to the anionic character of the oxygen atoms in the neutral NO_2 moiety. However, the frame at 12.5 ps indicated the formation of a ∧-shaped $[NO_2{\cdot}ArH]^+$ SET intimate complex. Spin density analysis at this stage revealed regeneration of the neutral NO_2 species, with positive spin density delocalized over the entire molecule.

Electronic properties at 23 ps, corresponding to a stabilized intermediate of the ∧-shaped $[NO_2{\cdot}ArH]^+$ SET complex, were consistent with those observed at 11.55 ps, once again supporting the notion that the electrophilic attack occurred through the oxygen atom of the nitronium ion due to its anionic characteristics. In the product frames (30 and 38 ps), both HOMO and LUMO orbitals were localized on the product molecules. The HOMO remained uniformly distributed across the NO molecule, while the LUMO showed greater localization on the carbonyl group of 2-methylcyclohexa-2,4-dien-1-one. This spatial distribution enabled strong interaction between the two products throughout

the simulation timeframe. Spin density results at 30 and 38 ps confirmed the presence of unpaired electrons on the oxygen atom of the NO molecule and across the structure of 2-methylcyclohexa-2,4-dien-1-one, particularly in the carbonyl group and delocalized over the conjugated ring, indicating the persistence of radical character in the final products.

Frame	SYSTEM	HOMO	LUMO	SPIN DENSITY
2 ps	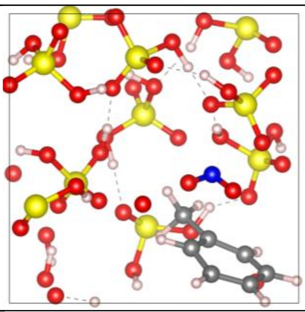	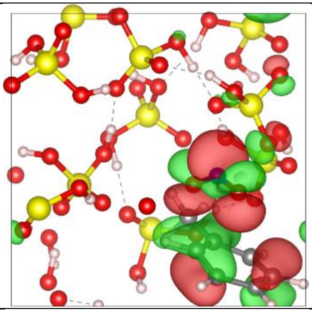	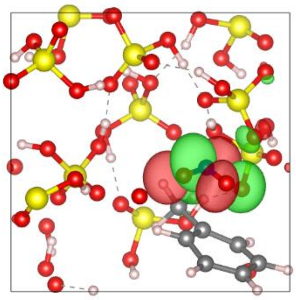	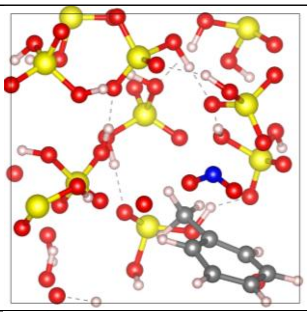
7 ps	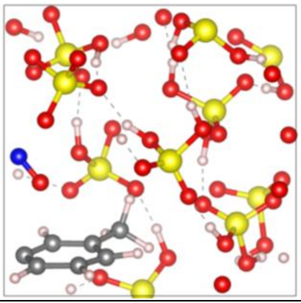	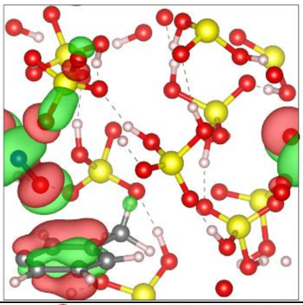	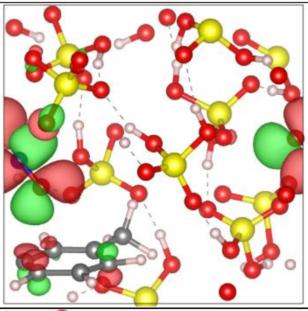	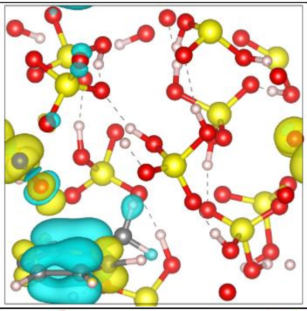
10 ps	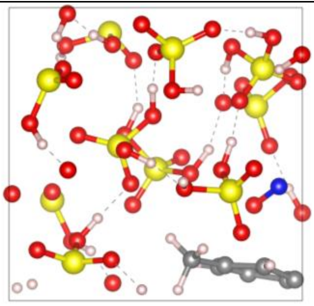	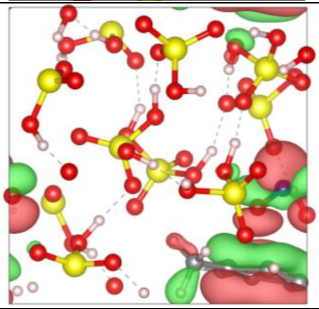	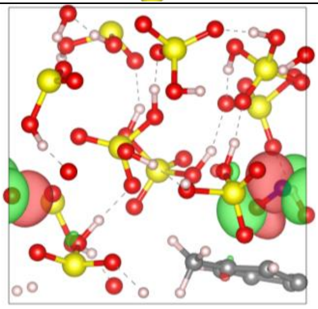	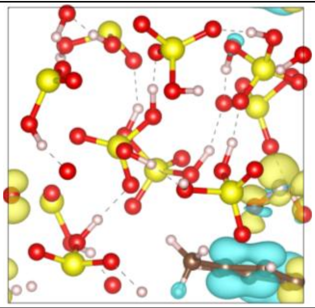
11.5 ps	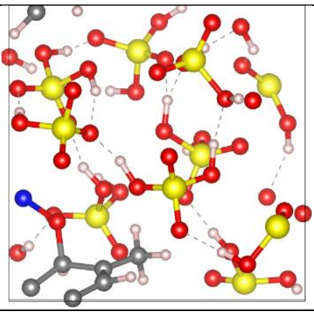	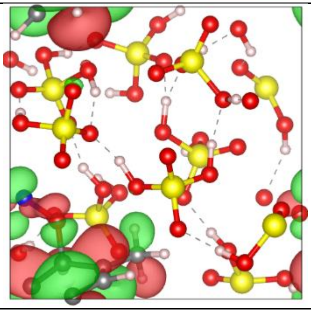	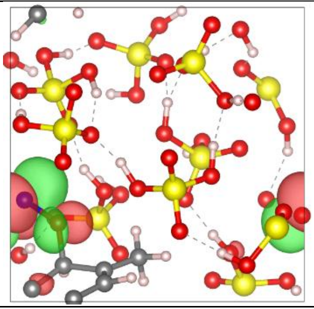	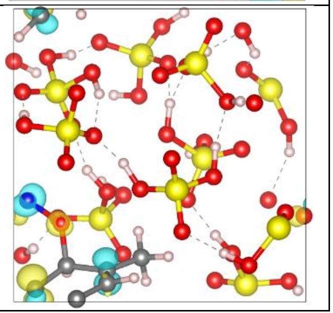

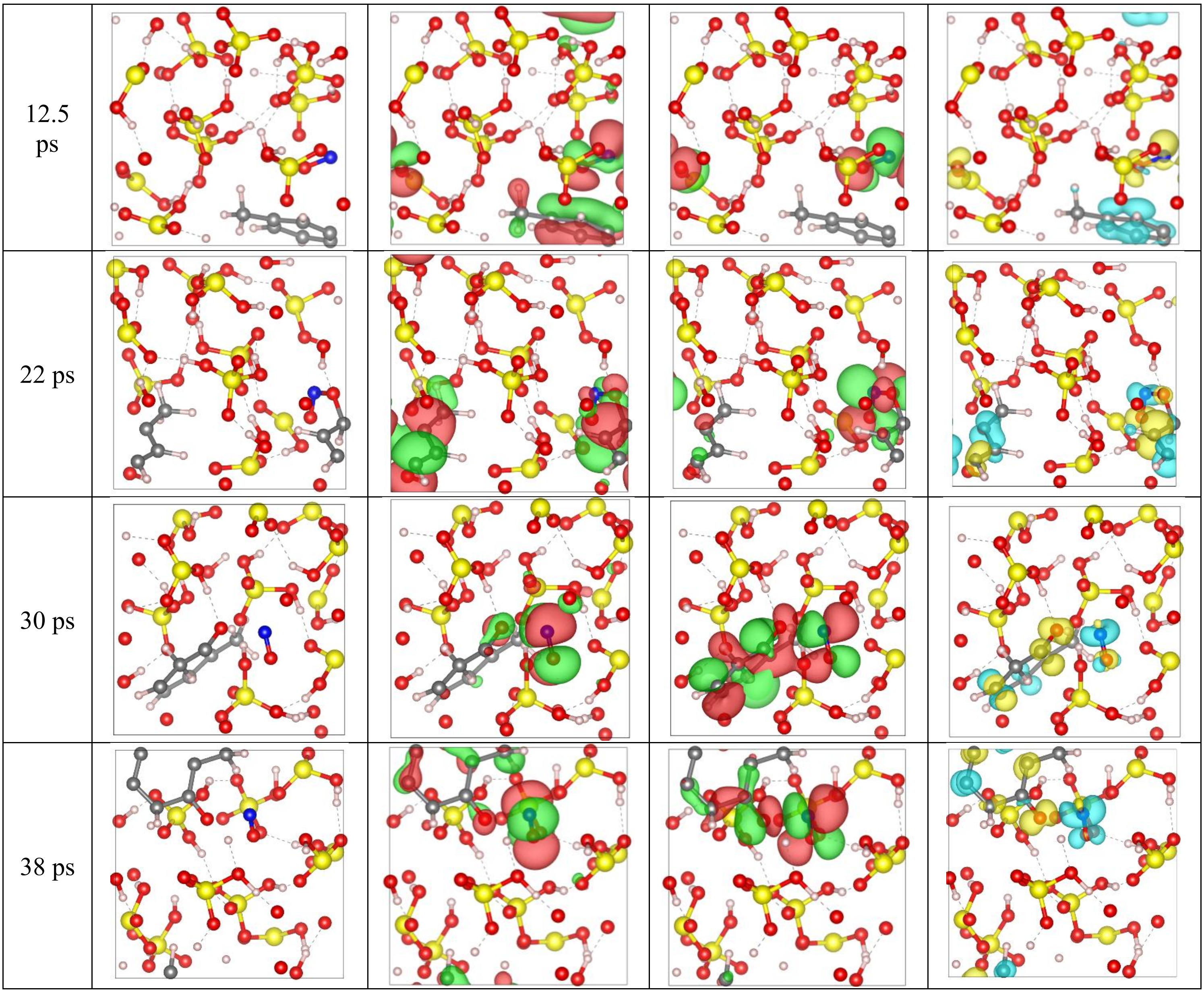

Figure S7. HOMO and LUMO orbitals, and spin density maps at representative time frames from the cyclohexadienone–NO complex formation simulation at 300 K. Atom color scheme: red = oxygen, yellow = sulfur, blue = nitrogen, gray = carbon, white = hydrogen. For HOMO and LUMO maps, red indicates positive orbital density and green indicates negative density. For spin density maps, yellow represents positive spin density and blue indicates negative spin density. An isosurface value of 0.025 was used for HOMO and LUMO, and 5×10^{-9} for spin density in all cases. All calculations were performed using the PBE functional and the cc-pVTZ basis set.

The Bader partial charges calculated for the selected frames of the methylcyclohexadienone–NO complex formation simulation are summarized in Table S3. At 2 ps, the nitronium ion withdrew electronic density from toluene, resulting in a net positive charge on the aromatic ring, more pronounced than in the early stages of the para-nitration and *o*-cresol simulations (see Tables 1 and S2). At 7 and 10 ps, the partial charges (+0.23*e* for NO_2 and +0.62*e* for toluene at 10 ps), combined with the spin density distributions shown in Figure S7, evidenced the presence of unpaired electrons, confirming the occurrence of a single electron transfer (SET) event.

At 11.5 ps, corresponding to the formation of the σ-complex, the nitro group exhibited a partial charge of -0.35*e*, while the toluene moiety showed a strongly positive charge of +1.29*e*, reflecting the electron-withdrawing nature of the NO_2 group. By 12.5 ps, the partial charges for NO_2 and toluene remained like those at 7 and 10 ps. Along with the spin density profile observed in Figure S7, these results suggest the dissociation of a neutral NO_2 species accompanied by the presence of unpaired electrons.

At 22 ps, which corresponds to the σ-complex formed after the second nucleophilic attack via the NO_2 oxygen, the calculated partial charges were -0.72*e* for NO_2 and +1.67*e* for toluene, values even more pronounced than those observed at 11.5 ps, indicating a more stabilized intermediate. Finally, in the product frames, both species exhibited positive partial charges, with 2-methylcyclohexa-2,4-dien-1-one slightly more positive (+0.5*e*) compared to the NO molecule (+0.4*e*). These values are consistent with the persistence of radical character in both products.

Table S3. Partial charges of the nitronium ion and toluene at selected time frames (3 ps, 7 ps, 10 ps, and 11.5 ps, 12.5 ps and 22 ps) during the cyclohexadienone–NO complex formation simulation. Also shown are the partial charges of the final products, 2-methylcyclohexa-2,4-dien-1-one and NO, at 30 ps and 38 ps. Unity of charge is *e*, the modulus of the electron charge ($e = 1.60217663 \times 10^{-19}$ coulombs). All calculations were performed using the PBE functional and the cc-pVTZ basis set.

**Reactant**	**2 ps**	**7 ps**	**10 ps**	**11.5 ps**	**12.5 ps**	**22 ps**	**Product**	**30 ps**	**38 ps**
**q(NO_2)**	+0.49	+0.23	+0.17	-0.35	+0.22	-0.72	**q(NO)**	+0.42	+0.35
**q(Toluene)**	+0.32	+0.62	+0.60	+1.29	+0.74	+1.67	**q(2-*methylcyclohexa-2,4-dien-1-one*)**	+0.50	+0.56
